# Development and validation of focal adhesion-related genes signature in gastric cancer

**DOI:** 10.3389/fgene.2023.1122580

**Published:** 2023-03-08

**Authors:** Guanghui Zhao, Tianqi Luo, Zexian Liu, Jianjun Li

**Affiliations:** ^1^ Department of Endoscopy, Sun Yat-sen University Cancer Center, State Key Laboratory of Oncology in South China, Guangzhou, China; ^2^ Department of Musculoskeletal Oncology, Sun Yat-sen University Cancer Center, State Key Laboratory of Oncology in South China, Guangzhou, China; ^3^ State Key Laboratory of Oncology in South China, Collaborative Innovation Center for Cancer Medicine, Sun Yat-sen University Cancer Center, Guangzhou, China

**Keywords:** gastric cancer, TCGA, focal adhesion, prognosis, GEO

## Abstract

**Background:** This study aims to build a focal adhesion-related genes-based prognostic signature (FAS) to accurately predict gastric cancer (GC) prognosis and identify key prognostic genes related to gastric cancer.

**Results:** Gene expression and clinical data of gastric cancer patients were sourced from Gene Expression Omnibus and The Cancer Genome Atlas. Subsequently, the GEO dataset was randomly distributed into training and test cohorts. The TCGA dataset was used to validate the external cohort. Lasso Cox regression was used to detect OS-related genes in the GEO cohort. A risk score model was established according to the screened genes. A nomogram, based on the clinical characteristics and risk score, was generated to predict the prognosis of gastric cancer patients. Using time-dependent receiver operating characteristic (ROC) and calibration performances, we evaluated the models’ validity. The patients were grouped into a high- or low-risk group depending on the risk score. Low-risk patients exhibited higher OS than high-risk patients (entire cohort: *p* < 0.001; training cohort: *p* < 0.001, test cohort: *p* < 0.001). Furthermore, we found a correlation between high-risk gastric cancer and extracellular matrix (ECM) receptor interaction, high infiltration of macrophages, CD44, and HLA-DOA.

**Conclusion:** The generated model based on the genetic characteristics of the focal adhesion prognostic gene can aid in the prognosis of gastric cancer patients in the future.

## Introduction

Gastric cancer (GC) is one of the most common solid tumors worldwide, responsible for 7.7% of all cancer-related deaths and ranking second only to lung and liver cancers (Sung et al., 2021). Unfortunately, early-stage GC is often asymptomatic, resulting in most patients being diagnosed at advanced stages (Sitarz et al., 2018).

Consequently, an accurate understanding of the heterogeneity of GC is crucial for predicting prognosis and tailoring clinical diagnosis and treatment. Focal adhesion is a complex of proteins that physically connects the extracellular matrix to the actin cytoskeleton and comprises several proteins, including integrins, cofilin proteins, and focal adhesion kinase (FAK) (Paluch et al., 2016). By regulating cell adhesion, migration, and differentiation, focal adhesion is essential for normal physiological functions, and its dysregulation can lead to tumorigenesis and metastasis (Damiano et al., 1999; Landowski et al., 2003). Focal adhesion has been found to upregulate B3 and FAK expressions in GC, which can facilitate cancer cells to resist fluorouracil, leading to treatment failure (Ngabire et al., 2020). In addition, focal adhesion proteins have been shown to promote GC cell invasion by enhancing cell proliferation (Shen et al., 2013). Therefore, inhibiting the focal adhesion signal pathway could potentially lead to effective treatment for GC.

Based on the univariate Cox regression analysis, significant genes associated with prognosis were identified, and a prognostic signature was constructed using Lasso analysis. ROC and Kaplan-Meier (KM) analyses were used to evaluate the performance of the signature.

Next, we constructed a nomogram based on the FAS and corresponding clinical characteristics. Finally, we validated the accuracy of our newly developed nomogram that predicts the prognosis of GC patients using an external validation cohort from TCGA. Overall, our study highlights the crucial role of focal adhesion-related genes in GC prognosis and presents a novel nomogram for predicting OS in GC patients.

## Materials and methods

### Data collection

The clinicopathological information and corresponding gene expression data of GC patients were obtained from the GEO database (http://www.ncbi.nlm.nih.gov/geo/). A total of 684 cases (GSE13861, GSE29272, GSE62254, and GSE26942) were examined in the entire cohort. 330 TCGA-STAD samples and their corresponding clinicopathological data were extracted from the TCGA (https://portal.gdc.cancer.gov/) to be used for the external validation cohort. A list of focal adhesion-related genes was retrieved from the MSigDB database (https://www.gsea-msigdb.org/gsea/msigdb) to aid this analysis.

### Data processing

Gene symbols for each gene matrix file were extracted based on the corresponding platform file used by the Perl software. The batch effect was adjusted using the Empirical Bayes method (“sva” package) among the series. Finally, the entire cohort was randomly divided into a training and a test cohort in a 7:3 ratio.

### FAS construction and validation

A total of 199 focal adhesion-related genes were selected from the MSigDB database. The relationship between these genes and gastric cancer prognosis was investigated through univariate Cox, LASSO regression, and multivariate Cox analyses using R packages “glmnet” and “survival.”

We calculated the risk score of every patient using the regression coefficient value and expression of each gene, as follows: = Σβi × Expi, where Expi refers to the gene expression level of the focal adhesion-related genes, and *β* is the LASSO Cox regression coefficient of the corresponding gene. Based on their risk scores, samples were categorized as high- or low-risk. The prediction accuracy of the signature was evaluated using time-dependent ROC curves and Kaplan-Meier survival analysis.

### Establishment and evaluation of a FAS-based nomogram model to predict OS of GC patients

Based on the focal adhesion risk score, the patient’s gender, age, and American Joint Committee on Cancer AJCC stage, a predictive nomogram was developed to accurately predict the OS of GC patients. The precision of the nomogram was evaluated using calibration and receiver operating characteristic (ROC) curves.

### Gene set enrichment analysis

The GSEA program was obtained from the GSEA website (http://www.gseamsigdb.org/gsea/index.jsp) to identify the functional enrichment pathways regulated by the focal adhesion -related genes signature. Additionally, the “c2.cp.kegg.v7.4.symbols.gmt” and “c5.go.v7.4.symbols.gmt” gene sets were extracted from the molecular signatures database and used as the target enrichment sets for GSEA analysis.

### Calculations of the immune, stromal, and estimate scores

The Estimation R package was used to estimate the immune and stromal component scores in the tumor microenvironment (TME) of each GC sample. The immune and stromal scores were then calculated and displayed, along with an estimated score. A positive correlation between the risk score and the immune, stromal, and overall scores suggests a higher risk score and a greater proportion of corresponding TME components.

The R packages “survival” and “survminer” were used to analyze the TME scores. A total of 684 samples with available survival data were divided into high- and low-scoring groups based on the median values of the immune, stromal, and estimated scores for subsequent survival analysis.

### Single-sample gene set enrichment analysis (ssGSEA)

The single sample gene set enrichment analysis (ssGSEA) in the “gsva” R package quantifies the infiltration statuses of 16 immune cells and the activities of 13 immune-related pathways in the high- and low-risk 

### Drug sensitivity analysis

The drug sensitivity analysis was conducted using cellminer (https://discover.nci.nih.gov/SclcCellMinerCDB/) Database data, screening FDA-approved and clinical trial data, and analyzing the relationship between focal adhesion-related genes expression level and drug sensitivity. Spearman’s correlation analysis was conducted to determine the correlation using R software, and the top 16 drugs were selected.

### Validation of a FAS-Based prognostic model in a clinical sample

The accuracy of the results was further validated using the STAD data from the KM-plotter database (https://kmplot.com/).

### Validating the expression of focal adhesion-related genes using scRNA-seq data

Download the GSE112301 dataset from the GEO website and use the CreateSeuratObject function from the SeuratR package to create a Seurat object that contains the basic information of the single-cell dataset. Next, quality control is performed on the data, including filtering out low-quality cells, followed by reducing the dimensionality of the data using principal component analysis (PCA). Visualize the PCA results to better distinguish the differences between cells in different tissue. Finally, use the FindConservedMarkers function of Seurat to analyze the gene expression of GC samples and normal samples.

### Statistical analyses

The statistical analyses were conducted using R software version 4.0.0, and KM survival analysis assessed the differential OS durations between the high- and low-risk groups. *p* < 0.05 was set as the significance threshold.

## Results

### Patient characteristics and establishment of FAS

**FIGURE 1:**
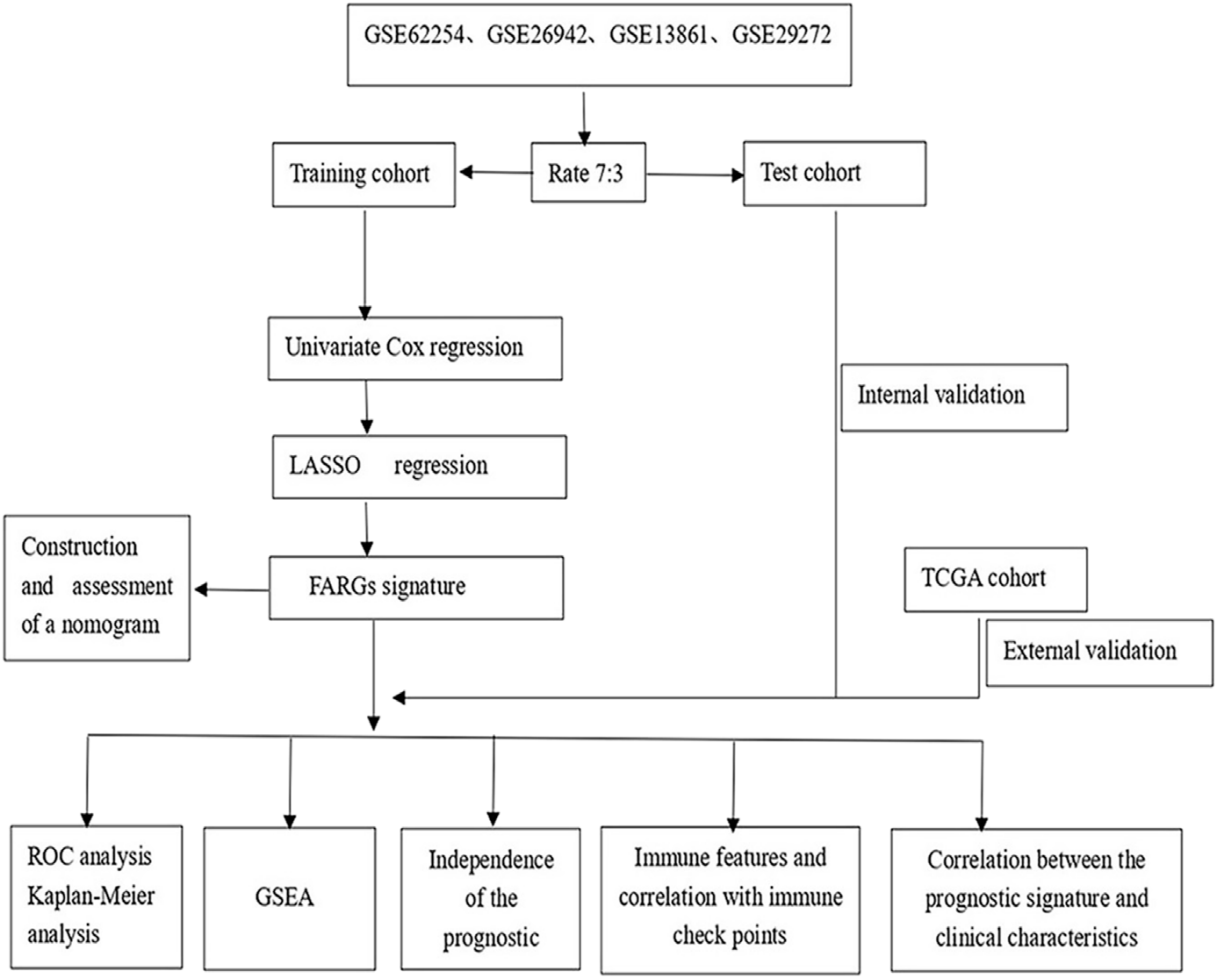
The fow chart showing the scheme of our study on focal adhesion prognostic signatures in GC.

**FIGURE 2:**
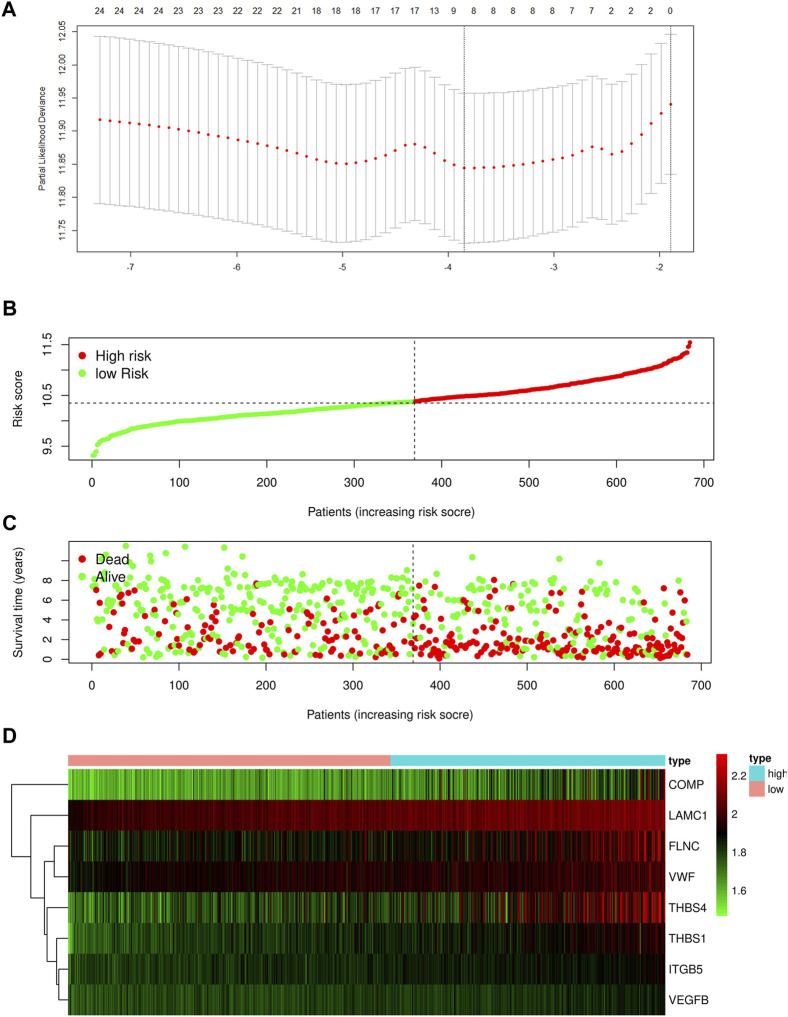
**(A)** Determination of the number of factors by the LASSO analysis. Risk score distribution **(B)**, survival status **(C)**, and eight focal adhesion-related genes expression profiles **(D)** for patients in high-risk and low-risk groups in training cohort.

As illustrated in Figure 1, upon exclusion of cases with a survival time of fewer than 30 days and normal cases, 684 samples were collated in the four GEO datasets (GSE13861, GSE26942, GSE29272, and GSE62254). These cases were randomly divided into a training (478) or test cohort (206) in a 7:3 ratio. Table 1 summarizes the patient clinical characteristics. The “limma” package extracted genes associated with focal adhesion in the GEO database. In the training cohort, the univariate Cox and LASSO regression analyses were conducted to screen eight genes associated with GC patient OS, as depicted in Figure 2A. The risk score was calculated according to a linear combination of the expression levels of the eight focal adhesion-related genes and corresponding regression coefficients (Table 2).

**TABLE 1:** Clinicopathological characteristics.

		Training cohort	478	Test cohort	206
Variable		High-risk	Low-risk	High-risk	Low-risk
N		244	234	111	95
Risk score (median)		14.61	13.96	14.65	13.93
Age (median)		61	61	60	63
Gender	Male	175	162	74	68
	Female	69	72	37	27
Stage	I	24	49	7	19
	II	44	51	21	33
	III	150	121	73	40
	IV	26	13	10	3
Overall survival	Alive	105	168	50	66
	Dead	39	66	61	29
Survival time (median)		804	1807	881	1821

**TABLE 2:** Details of the eight focal adhesion-related genes in the prognostic model.

Gene name	Coefficient	HR	HR.95L	HR.95H	*p* value
COMP	0.329464	2.436025	1.552258	3.822958	0.000108
FLNC	0.254581	2.802215	1.614737	4.862965	0.000249
ITGB5	0.77376	39.25463	9.107316	169.1965	8.50E-07
LAMC1	0.896998	16.40379	6.121545	43.95691	2.66E-08
THBS1	0.507687	7.57718	3.451558	16.63413	4.47E-07
THBS4	0.015673	2.370891	1.678713	3.348471	9.54E-07
VEGFB	0.836806	16.9152	4.07668	70.18554	9.79E-05
VWF	0.249564	7.56926	2.922742	19.60272	3.06E-05

Risk score = CMOP × (0.3294) + FLNC × (0.2545) + ITGB5 × (0.7737) + LAMC1 × (0.8969) + TBHS1 × (0.5076) + THBS4 × (0.0156) + VEGFB × (0.8368) + VWF × (0.2495).

Based on the optimal cut-off value of the risk score determined using the Survminer R package, the patients were categorized into either a high- or low-risk group. Further independent prognostic analysis of all the key genes revealed a significant association between high expression levels of these genes and poor prognosis in the training cohort (Supplementary Figure S1).

**FIGURE 3:**
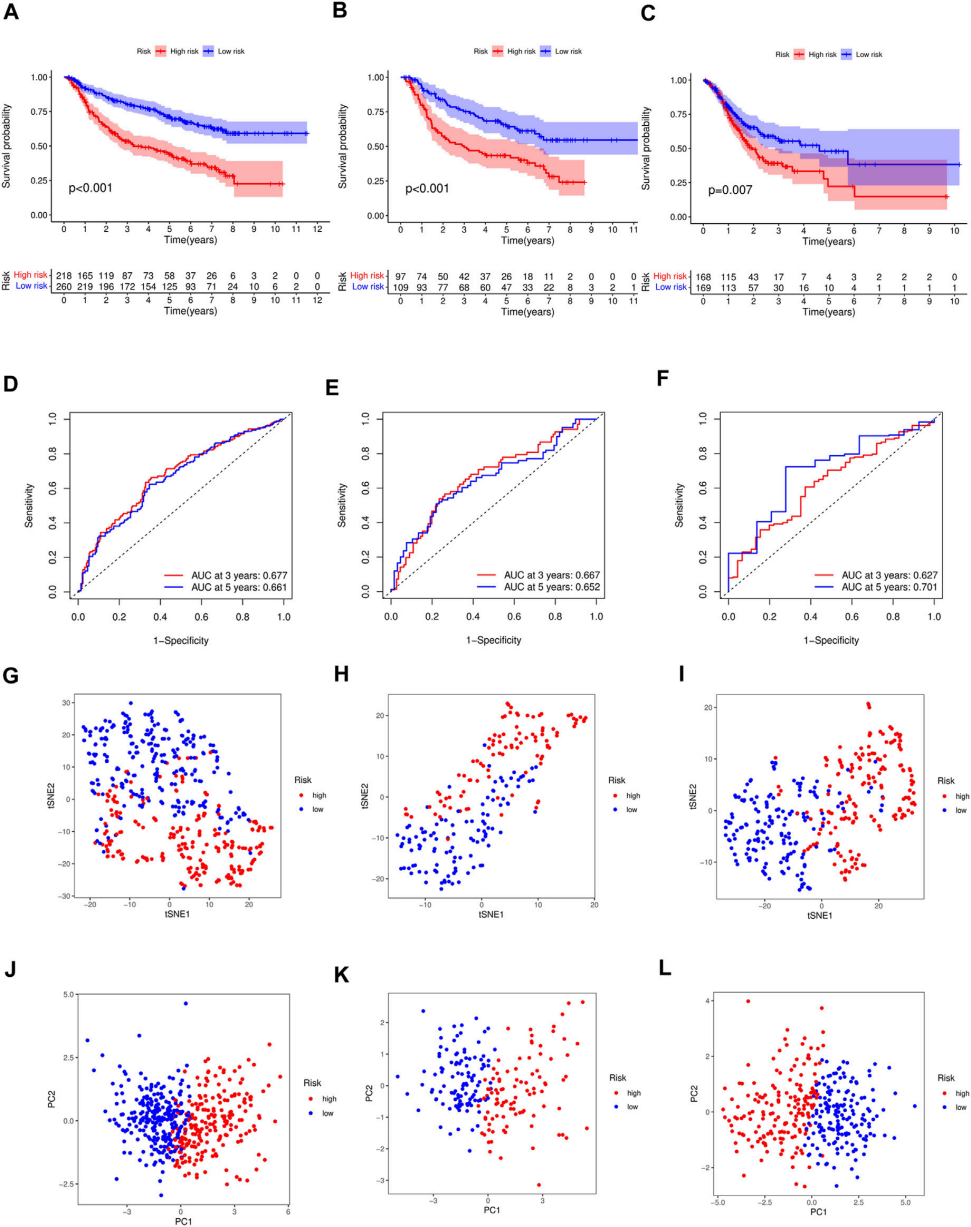
**(A)** Differences in survival between high-risk and low-risk groups of the training cohort (*p* < 0.001). Differences in survival between high-risk and low-risk groups of the test cohort (*p* < 0.001) **(B)** and external cohort (*p* =0.007) **(C)**. **(D)** Time-dependent ROC analysis of the FAS in the training cohort. **(E)** Time-dependent ROC analysis of the FAS in the test cohort. **(F)** Time-dependent ROC analysis of the FAS in the external cohort. **(G)** The t-SNE plot in the training cohort. **(H)** The t-SNE plot in the test cohort. **(I)** The t-SNE plot in the external cohort. **(J)** The PCA plot in the training cohort. **(K)** The PCA plot in the test cohort. **(L)** The PCA plot in the external cohort.

The distribution of the risk scores and the survival statuses of patients in the training cohort are displayed in Figures 2B–D. KM analysis determines the differences between the two groups in the training cohort (Figure 3A). Lastly, time-dependent ROC exhibits the prognostic values of our signature (Figure 3D).

### Validation and evaluation of the prognostic gene signature

Test and external validations were conducted. Consistent with the training cohort results, OS was lower in high-risk patients than in low-risk patients (Figure 3B, *p* < 0.001 in test cohort; Figure 3C, *p* = 0.007 in TCGA cohort). The area under the ROC curve demonstrated that the signature could precisely predict GC prognosis (Figures 3E, F). The AUCs for the test and external validation cohorts were 0.667 and 0.627 at 3 years, respectively, and 0.652 and 0.701 at 5 years, respectively. The accuracy of the signature was evaluated using PCA and t-SNE analyses. Both PCA and t-SNE plots revealed that the high- and low-risk groups had different directions in the training (Figures 3G, J), test (Figures 3H, K), and external cohorts (Figures 3I, L).

### FAS is an independent predictor of GC

**FIGURE 4:**
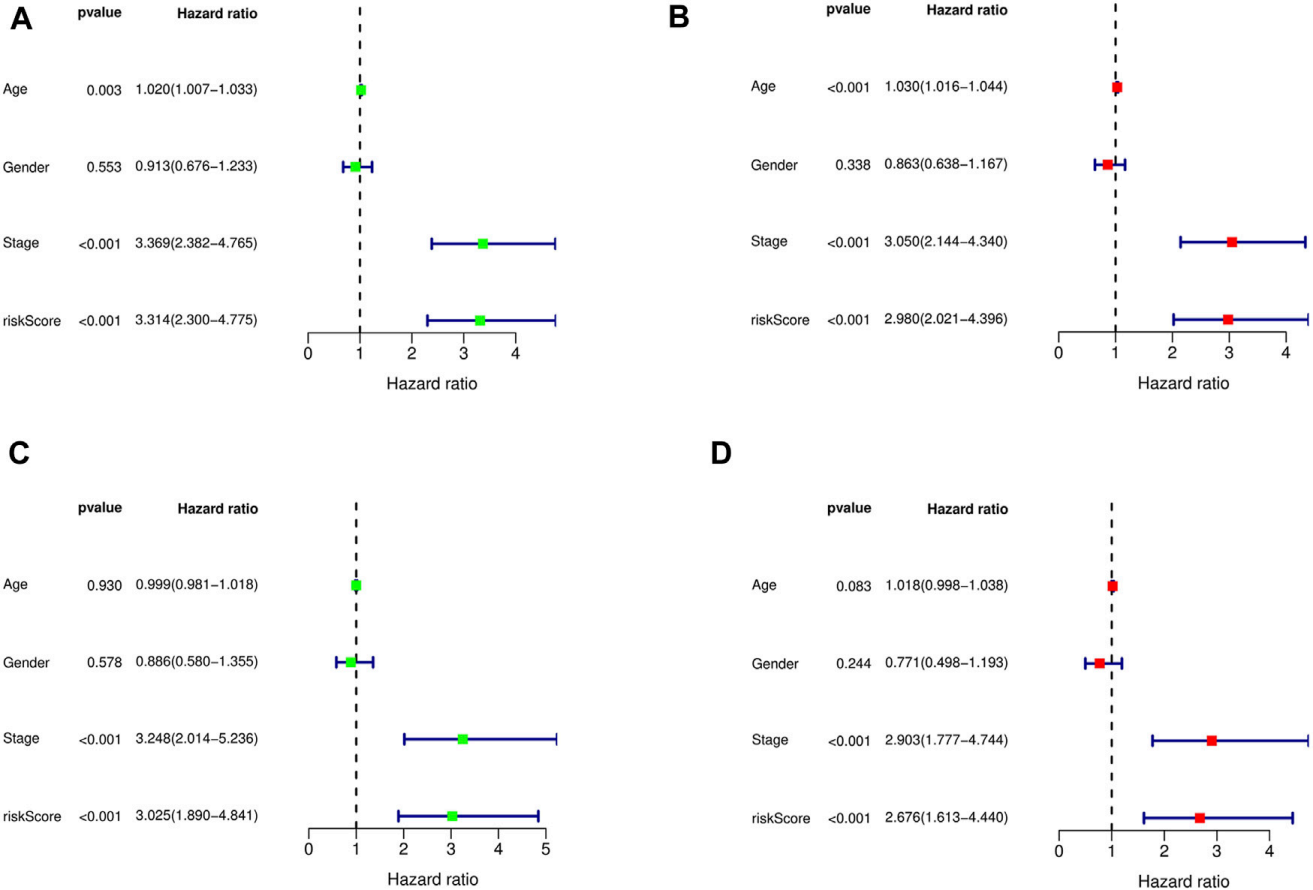
Univariate and multivariate Cox regression analysis showed the relationship between age, gender, stage, risk score, and overall survival, and indicated that risk score could be used as an independent prognostic factor for training cohort **(A,B)**, test cohort **(C,D)**.

The Cox regression analysis demonstrates the relationship between the risk scores acquired from the prognostic model with other clinical parameters. Based on the uni- and multivariate regression analyses, stage (*p* < 0.001, HR = 3.369; *p* < 0.001, HR = 3.050, respectively) and risk score (*p* < 0.001, HR = 3.314; *p* < 0.001, HR = 2.980, respectively) were independent OS prognostic factors in the training cohort (Figures 4A, B). The test cohort was validated, whereby both stage (*p* < 0.001, HR = 3.248; *p* < 0.001, HR = 2.903, respectively) and risk score (*p* < 0.001, HR = 3.025; *p* = 0.002, HR = 2.676, respectively; Figures 4C, D) were demonstrated to be independent risk factors for OS in GC patients.

### Subgroup analysis of the prognostic value of FAS

**FIGURE 5:**
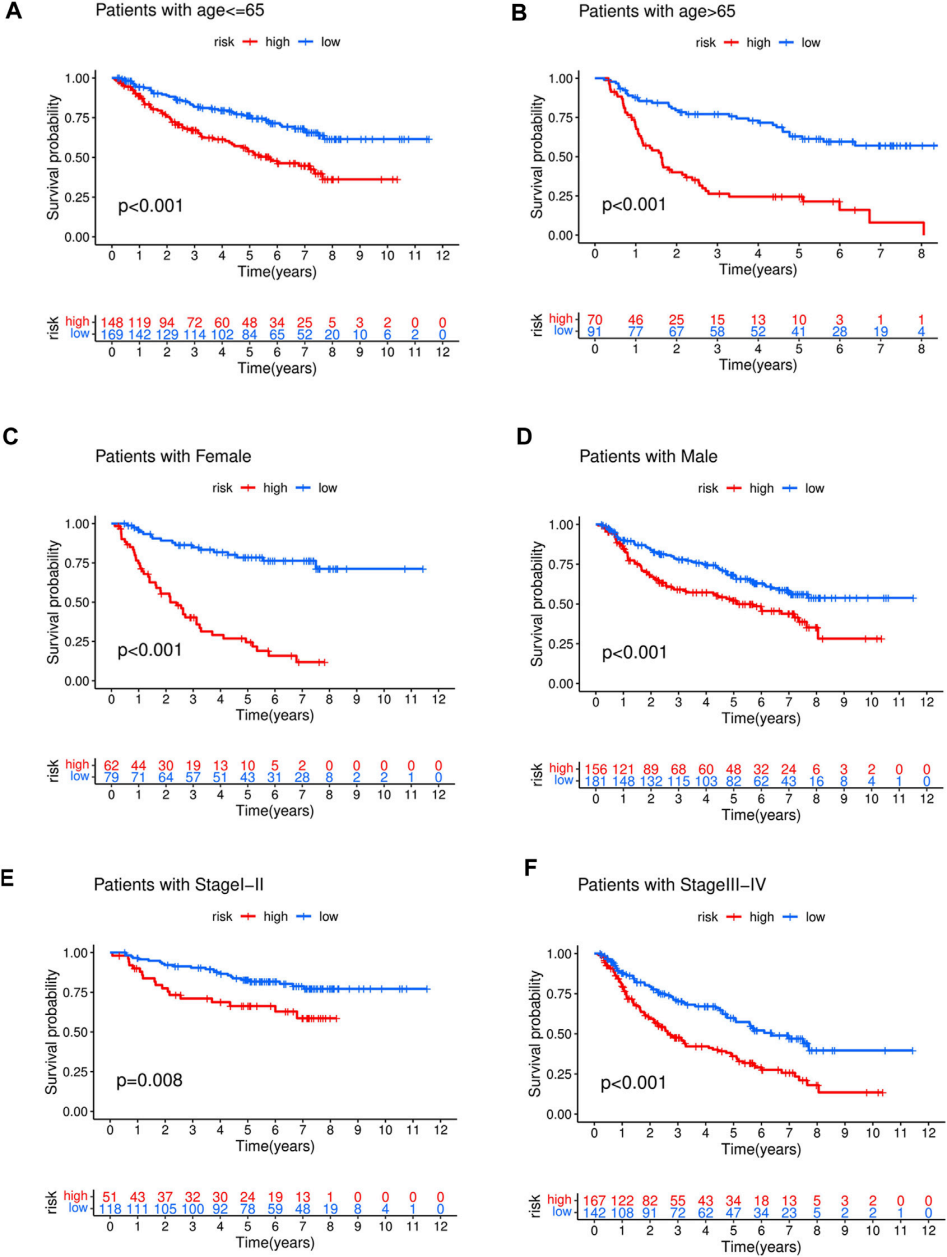
The high-risk group in training cohort showed a poor prognosis than the low-risk group in different clinical stratification like age **(A,B)**, gender **(C,D)**, stage **(E,F)**.

To investigate the prognostic value of the developed model in different patient populations based on their clinical characteristics, the training cohort was further divided into subgroups, and OS was estimated between high- and low-risk groups in each subgroup. The KM analysis showed that the risk score could distinguish differences between various subgroups, such as age, gender, and stage (Figures 5A–F). Similarly, in the test cohort, the high- and low-risk groups demonstrated differences in age and gender, but no significant differences were observed in the stage I-II subgroup, which may be attributed to the relatively small sample size in our study (Supplementary Figures S2A–F).

**FIGURE 6:**
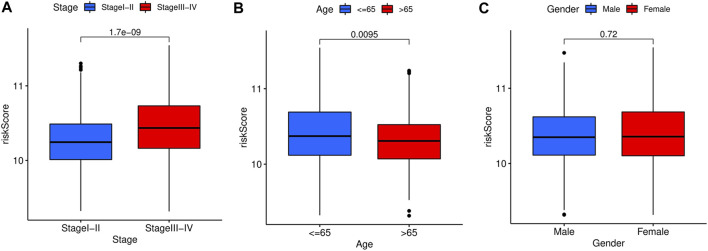
Stratified analysis of the prognostic signature in the training cohort. The relationships between the FAS and age **(A)**, gender **(B)**, stage **(C)**.

The relationship between risk score and corresponding clinical characteristics of GC patients (*n* = 684) in the GEO dataset was analyzed. The risk scores of stage I + II patients were significantly lower than those of stage III + IV patients (*p* = 1.7e-09, Figure 6A). Similar results were shown in the age subgroup (*p* = 0.0095, Figure 6B). No significant relationship between the risk score and gender (*p* = 0.72, Figure 6C).

### Generation of a prognostic nomogram that predicts OS in GC patients

**FIGURE 7:**
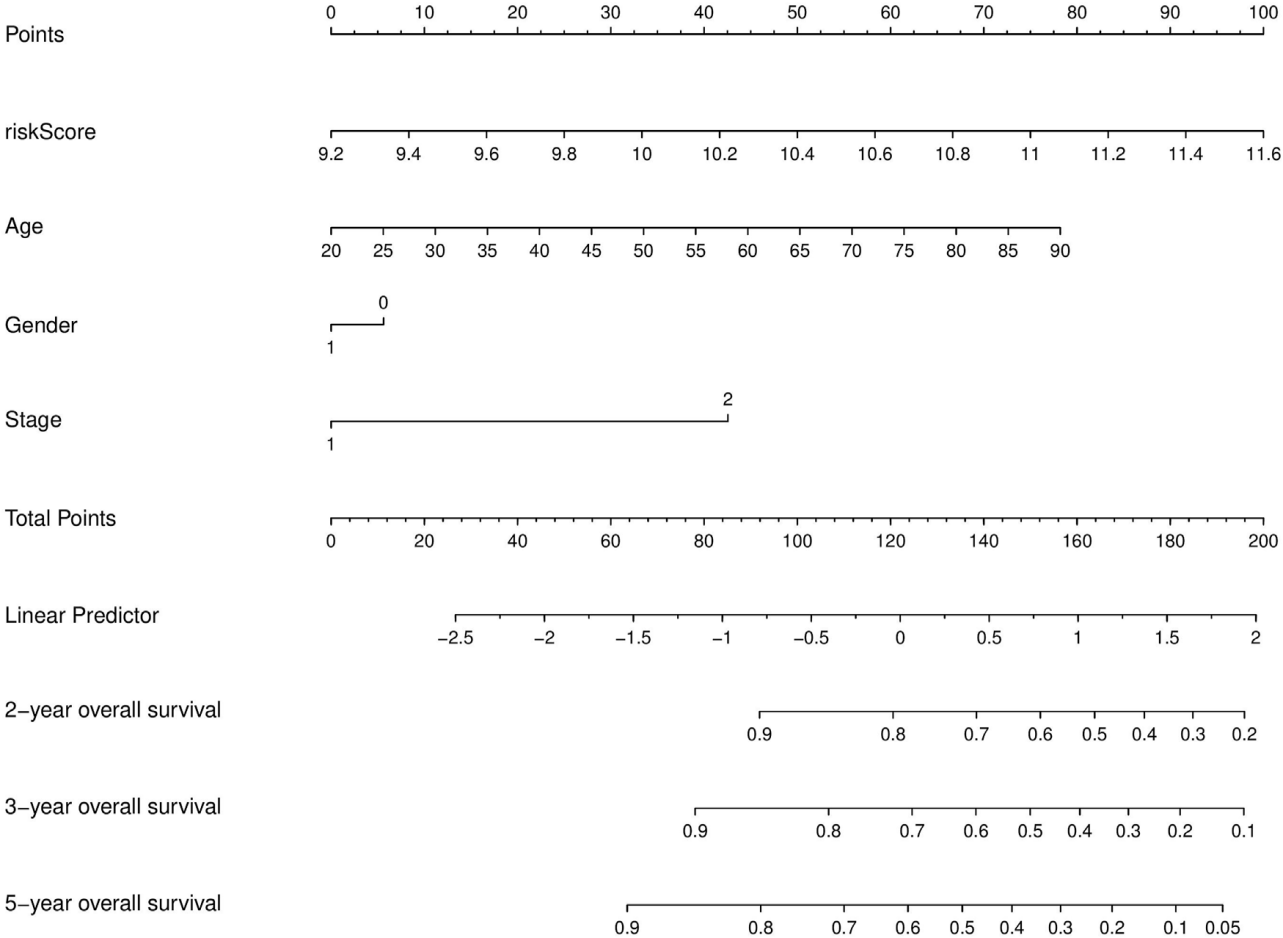
Nomogram for the prediction of 1-, 3-, and 5-year survival probability in patients GC.

**FIGURE 8:**
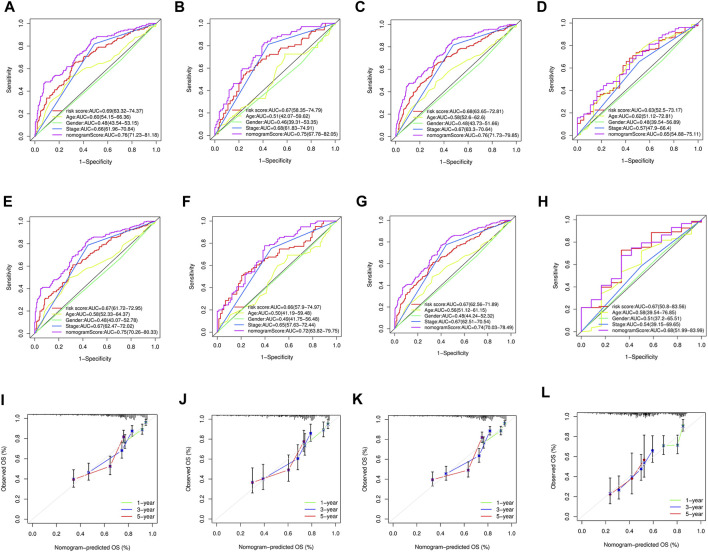
**(A–D)** 3 years time-dependent ROC analysis for nomogram in GC. **(A)** Time-dependent ROC analysis of the nomogram in the training cohort. **(B)** Time-dependent ROC analysis of nomogram in the test cohort. **(C)** Time-dependent ROC analysis of the nomogram in the entire cohort. **(D)** Time-dependent ROC analysis of the nomogram in the external cohort. **(E–H)** 5 years time-dependent ROC analysis for nomogram in GC. **(E)** Time-dependent ROC analysis of the nomogram in the training cohort. **(F)** Time-dependent ROC analysis of nomogram in the test cohort. **(G)** Time-dependent ROC analysis of the nomogram in the entire cohort. **(H)** Time-dependent ROC analysis of the nomogram in the external cohort. **(I)** The calibration plot for training cohort. **(J)** The calibration plot for test cohort. **(K)** The calibration plot for entire cohort. **(L)** The calibration plot for external cohort.

To accurately predict the prognosis of GC patients, a nomogram was developed based on uni- and multivariate regression analyses to predict 1-, 3-, and 5-year OS rates (Figure 7). Additionally, 3 years time-dependent ROC analysis revealed that the sensitivity of the nomogram was higher than other clinicopathological features in training cohort (Figure 8A), test cohort (Figure 8B), entire cohort (Figure 8C) and external cohort (Figure 8D). 5 years time-dependent ROC analysis for nomogram in training cohort (Figure 8E), test cohort (Figure 8F), entire cohort (Figure 8G) and external cohort (Figure 8H). The calibration plots for the training, test, entire cohort and external cohort were in agreement between the actual OS and the predicted from the nomogram (Figures 8I–8L).

The nomogram generated to predict the 1-, 3-, and 5-year OS rates of GC patients was found to be accurate, as evidenced by the 3-year and 5-year time-dependent ROC analysis and the calibration plots. These results suggest that the nomogram is a reliable tool for predicting the prognosis of GC patients.

### GSEA

**FIGURE 9:**
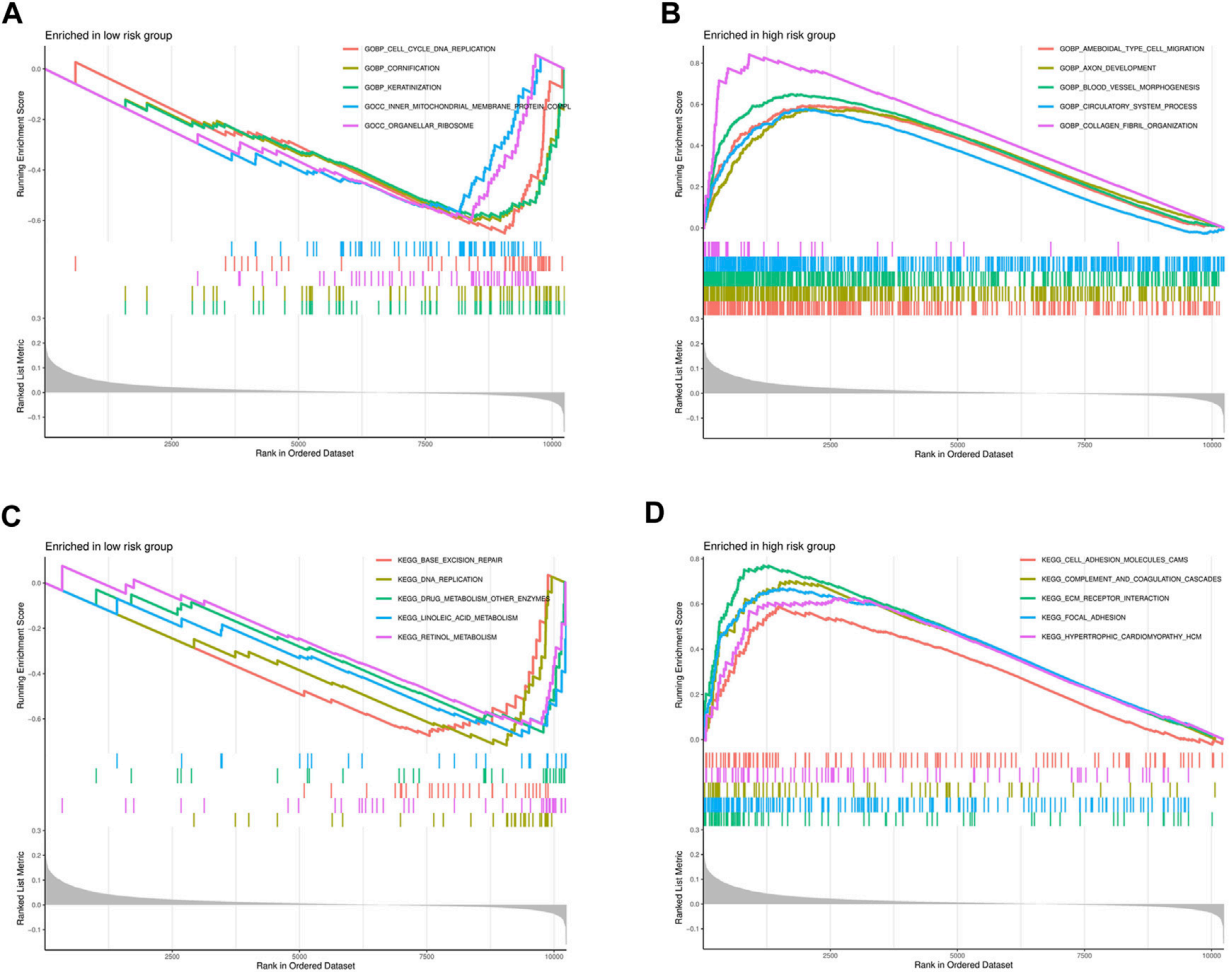
Significantly enriched GO pathways and KEGG pathways in the entire cohort by GSEA. **(A)** The GSEA analysis for GO pathway in the low-risk group. **(B)** The GSEA analysis for GO pathway in the high-risk group. **(C)** The GSEA analysis for KEGG pathway in the low-risk group. **(D)** The GSEA analysis for KEGG pathway in the high-risk group.

To explore the functional and signaling pathway differences between the high- and low-risk score groups, GSEA was performed on the gene sets “c5.go.v7.4.symbols.gmt” and “c2.cp.kegg.v7.4.symbols.gmt.” The top five pathways and gene functions in the high- and low-risk groups are displayed in Figures 9A–D.

### Immune cells infiltration and immune-related pathways

The tumor microenvironment (TME) plays a critical role in regulating tumor treatment resistance and is associated with tumor occurrence, development, and metastasis. It includes various components such as tumor cells, immune cells, stromal cells, and a variety of cytokines Changes in the TME, including alterations in the immune cell components, can promote tumor progression. To analyze the distribution of immune cells in the GC TME and investigate the interaction between GC tumors and immune cells, we utilized the ssGSEA tool to predict 16 common immune cells and 13 immune-related functional components based on GC gene expression profile data.

**FIGURE 10:**
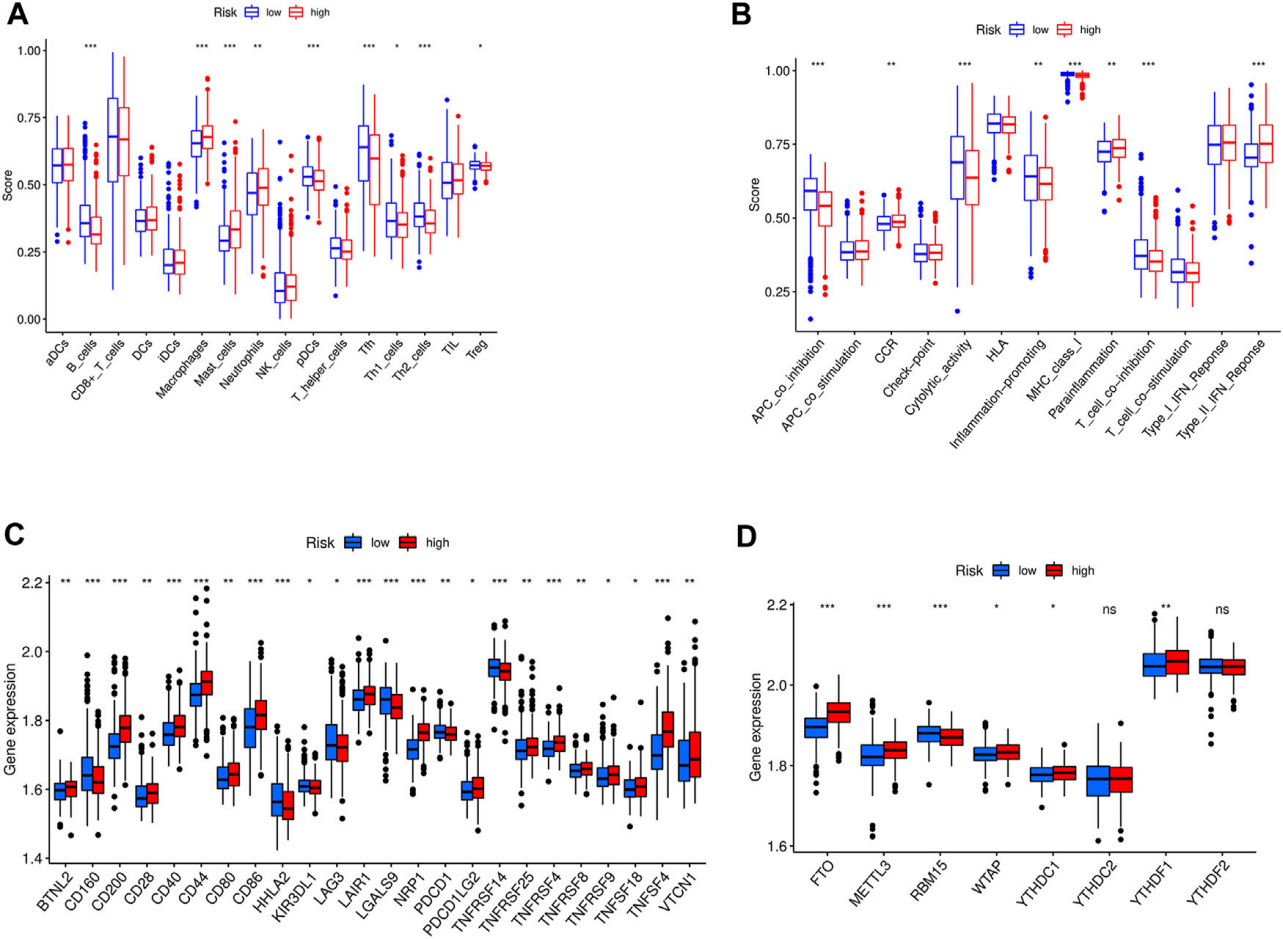
**(A)** The immune cell between high-risk and low-risk groups in entire cohort; ***p < 0.05, **p < 0.01, *p < 0.001; **(B)** The immune related function between high-risk and low-risk groups in entire cohort; ***p < 0.05, **p < 0.01, *p < 0.001; **(C)** The checkpoint differences between high-risk and low-risk groups in entire cohort; ***p < 0.05, **p < 0.01, *p < 0.001; **(D)** The m6A expression differences between high-risk and low-risk groups in entire cohort.***p < 0.05, **p < 0.01, *p < 0.001.

The low-risk group of patients showed higher ratios of B cells, T regulators, and follicular helper T cells than the high-risk group (Figure 10A). In addition, the low-risk group exhibited higher levels of antigen-presenting cell (APC) co-inhibition, inflammation promotion, and T cell inhibition compared to those with a high-risk score (Figure 10B). Additionally, we demonstrated that the expression levels of CD200, CD28, CD40, CD44, CD86, LAIR1, NRP1, TNFRSF4, TNFRSF8, TNFSF18, TNFSF4, and VTCN1 in the high-risk group were higher than those in the low-risk group (Figure 10C). The findings suggest that the immune microenvironment may be partly associated with the OS prognosis of GC patients with high expression of focal adhesion-related genes.

### Correlation of m6A expression

N6-methyladenosine (m6A) is the most abundant RNA modification in eukaryotic cells (Yue et al., 2015). Extensive RNA processing and metabolism research revealed that m6A is a key contributor to cancer development. m6A is a potential prognostic marker involved in multiple aspects of cancer treatment (Ma et al., 2019). To assess the relationship between m6A expression and our GC prognostic signature, the levels of 13 m6A genes in different GC samples were estimated. It is found that an elevated expression of FTO, METTL3, YTHDC1, and YTHDF1 genes in the high-versus low-risk group (Figure 10D).

### Correlation between TME subcomponents and the focal adhesion-related genes risk score and outcome of GC patients

**FIGURE 11:**
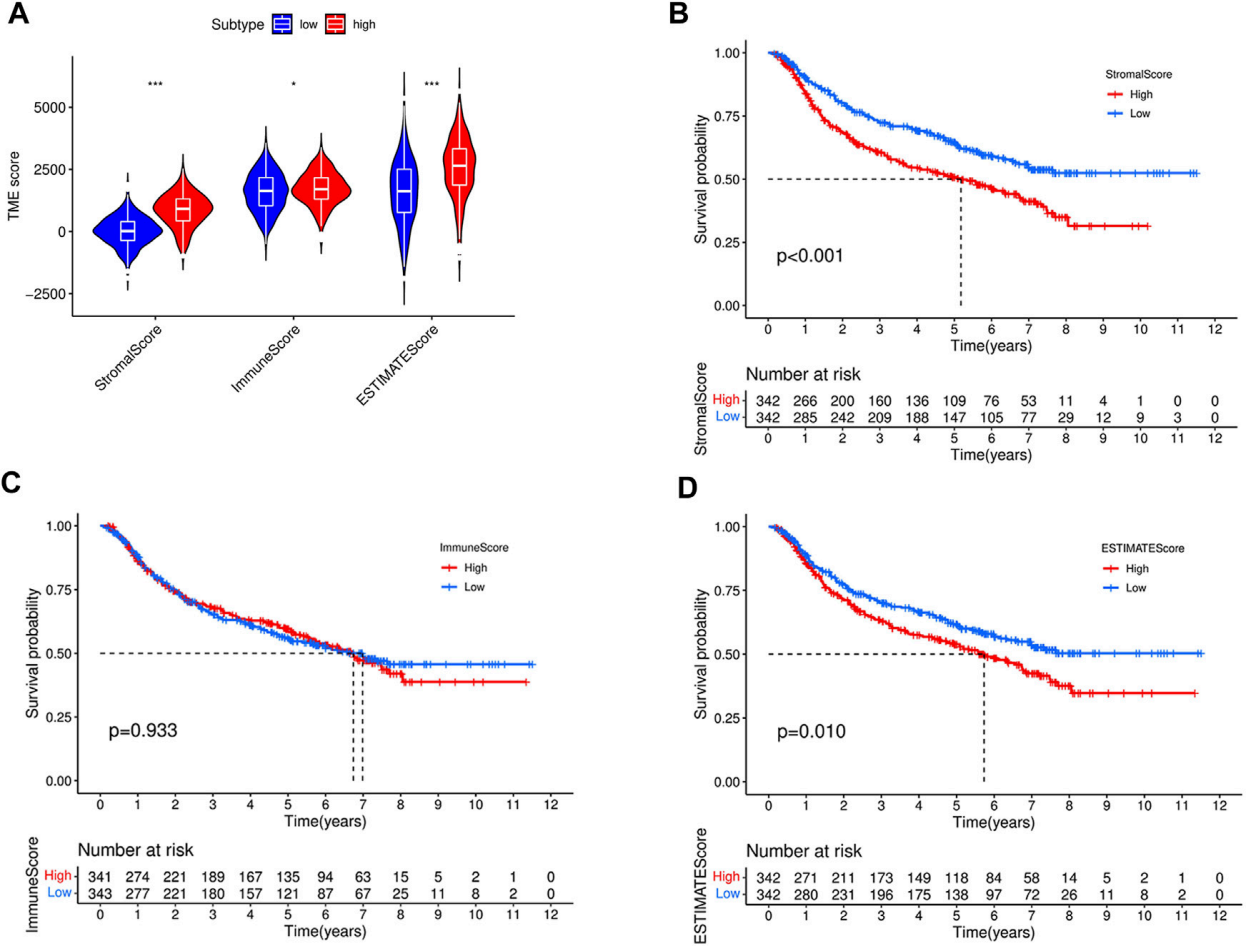
Comparison of stromal score, immune score, ESTIMATE scores, between high- and low-risk groups in the entire cohort **(A)**. **(B)** Kaplan–Meier curves for overall survival of 684 GC patients according to stromal score. Log-rank test, *p* < 0.001. **(C)** Kaplan–Meier curves for overall survival of 684 GC patients according to immune score. Log-rank test, *p* = 0.933. **(D)** Kaplan–Meier curves for overall survival of 684 GC patients according to ESTIMATE score. Log-rank test, *p* = 0.01.

TME consists of diverse immune and stromal cells linked to disease development, prognosis, and treatment outcome. Based on our ESTIMATE algorithm, TME was separated and scored into stromal, immune, and estimate subcomponents to investigate potential relationships between this study’s risk scores and TME. A high immune or matrix score indicates a high proportion of the immune or matrix components in the TME. The ESTIMATE score is the sum of the immune and stromal scores, indicating the combined proportion of these two components in TME. In our study, patients in the high-risk group in entire cohorts had higher stromal, immune, or ESTIMATE scores (Figure 11A) than those in the low-risk group.

**FIGURE 12:**
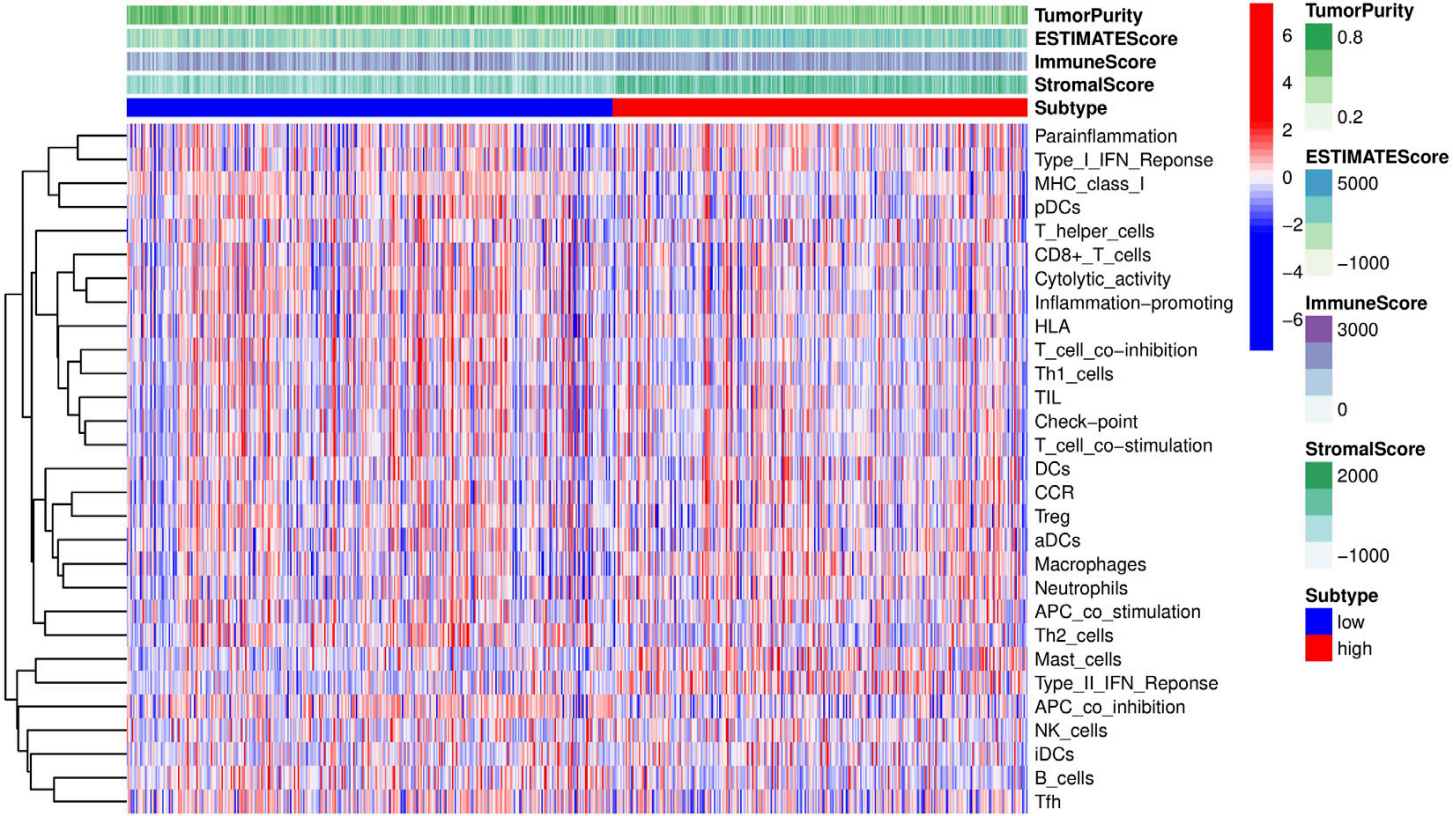
Heatmap revealing the scores of immune cells in the high-risk and low-risk groups.

To further investigate the impact of different components of the TME on GC patient survival, the entire cohort was divided into subgroups based on the median immune, stromal, and ESTIMATE scores as cutoff points. As shown in Figures 11B, D, patients with high stromal and ESTIMATE scores had worse overall survival than those with low stromal and ESTIMATE scores (*p* < 0.001, *p* = 0.01, respectively). However, the survival rate was similar between patients with high and low immune scores (*p* = 0.933) (Figure 11C). To better understand the association between the immune microenvironment and GC prognosis, a heatmap was generated to display the distribution of immune cell scores in the high- and low-risk groups, as shown in Figure 12.

### Drug sensitivity prediction

**FIGURE 13:**
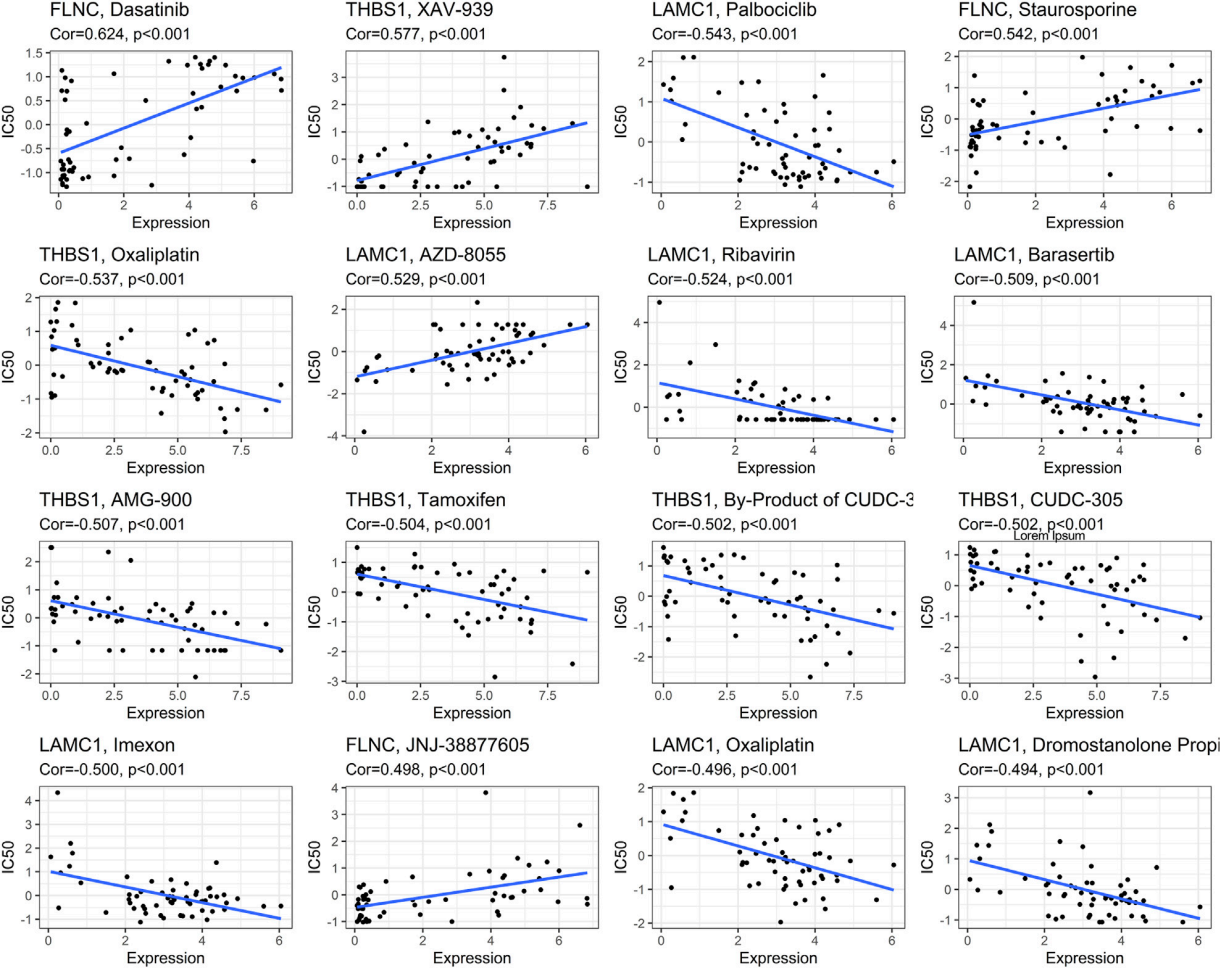
Correlations between focal adhesion-related genes expression and drug sensitivity. The figure shows the top 16 significant drug-gene pairs with significant correlation. *X*-axis: gene expression; *y*-axis: drug sensitivity Z scores.

The correlation between drug Z score and genes was analyzed, and the top 16 significant drug-gene pairs are displayed in Figure 13. A total of 246 drugs showed statistical differences, as shown in Supplementary Table S2. Among them, Dasatinib, XAV-939, and Staurosporine exhibited the most positive correlation with hub gene expression. In contrast, Palbocic, Oxaliplatin, and Ribavirin were negatively correlated with the expression of hub genes (Figure 13).

### Verification of a focal adhesion-based prognostic model in a clinical sample

**FIGURE 14:**
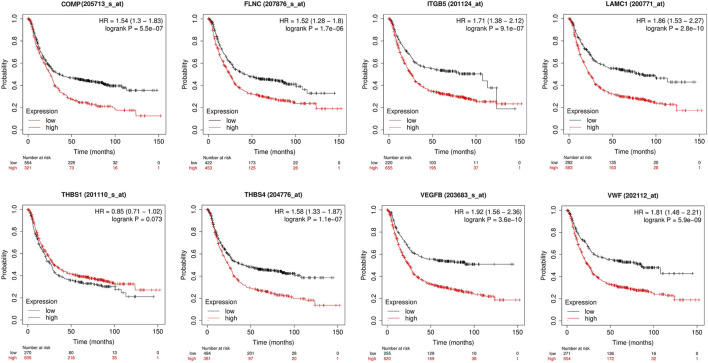
Univariate survival analysis of the focal adhesion-related genes using Kaplan-Meier curves.

To investigate the prognosis of patients with different hub genes expressions, the clinical data of STAD in the KM-plotter database were analyzed. Patients with high hub genes expression had better overall survival (OS) than those with low expression, except for THBS1 (Figure 14).

### Verification the expression of hub genes using scRNA-seq data

The Single-cell RNA sequencing dataset GSE112302 was used for further analysis in high resolution. A total of three GC and three normal samples were included in our study.

**FIGURE 15:**
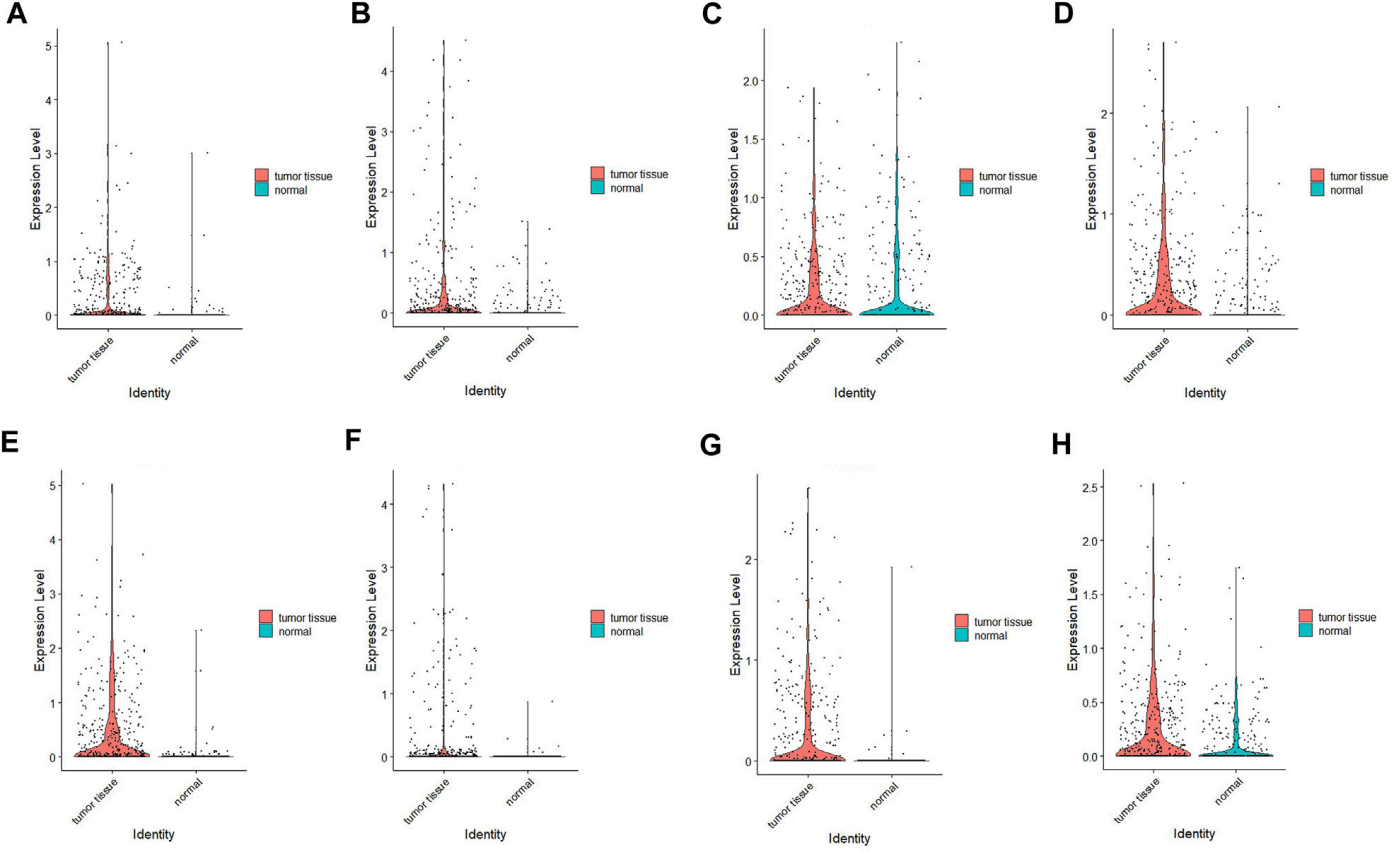
Verification results of four hub genes’ expression using scRNA-seq data. **(A)** COMP; **(B)** FLNC; **(C)** ITGB5, **(D)** LAMC1, **(E)** THBS1, **(F)** THBS4, **(G)** VEGFB, **(H)** VWF.

After quality control of the data, standardization and normalization were performed, followed by PCA and UMAP (Supplementary Figures S3A, B). A total of 305 normal cells and 401 tumor cells were included in the analysis. The UMAP plots of each gene in different tissues are displayed in Supplementary Figure S3C. Most genes exhibited high expression in tumor cells, except for ITGB5, which may be due to the limited sample size (Figure 15).

## Discussion

Patients with GC often do not experience symptoms in the early stages of the disease and may miss the opportunity for surgery due to local or distant metastasis at the time of diagnosis.

The development of the disease is influenced by multiple factors, and relying on a single factor or gene may not be a dependable prognostic marker. In this study, we systematically analyzed a group of genes related to patient survival and identified a strong association between the expression of focal adhesion-related genes and gastric cancer prognosis. The formation and turnover of focal adhesion are critical to tumor cell migration and progression (Eke and Cordes, 2015; Maziveyi et al., 2018; Wu et al., 2021). Therefore, evaluating the prognostic value of focal adhesion-related genes in GC patients is essential.

This study employed a bioinformatics approach and publicly available TCGA and GEO databases to identify focal adhesion-related genes. A risk score was assigned to each case to predict their prognosis, and high-risk cases were found to have a worse prognosis than low-risk ones. Furthermore, a nomogram was developed by combining risk scores and relevant clinical characteristics to demonstrate the accuracy of the prognostic model in predicting 3- and 5-year survival rates in GC patients. The focal adhesion-related genes identified in this study were highly predictive of GC prognosis and accurately characterized individual patient informationPrevious studies have established the association of the focal adhesion-related genes with cancer, specifically GC. In colon cancer, COMP levels were found to be significantly elevated, and were strongly associated with cell adhesion and tumor progression (Nfonsam et al., 2020). IGF1R signaling regulates the biological process of GC by increasing *β*-Catenin activation, epithelial-mesenchymal transition, and cell proliferation (Xu D et al., 2017). Yang et al. (2021) demonstrated that reducing the expression of ITGB5 using CRISPRa and CRISPRi technologies led to inhibition of cell proliferation. One prior study found that ITGB5 promotes lymph node metastasis in colorectal cancer patient (Capriotti et al., 2020). Another study by Han et al. (2021) showed that knockdown of LAMC1 inhibits GC cell proliferation, migration, invasion, and the Warburg effect by suppressing AKT and MEK/ERK pathways. Additionally, extracellular matrix proteins THBS1 and THBS4 strongly regulate key tumor cell processes, such as proliferation, attachment, adhesion, and migration. Elevated expressions of THBS1 and THBS4 may be closely associated with higher tumor grading and poorer prognosis in GC patients (Chen et al., 2019; Zhang et al., 2021). Filamin C is an essential component of the actin cytoskeleton and is encoded by the FLNC gene. As a member of the filamin family, it forms dimers and plays a crucial role in regulating cell motility, adhesion, and migration (Eden and Frey, 2021). In recent years, studies have found that the expression of FLNC is dysregulated in several types of cancer, including gastric cancer, glioma, liver cancer, and prostate cancer, and it is involved in tumor invasion and metastasis (Kokate et al., 2018; Kamil et al., 2019).

GO analysis was performed to identify the biological functions that are more relevant to high-risk patients. The analysis revealed that high-risk patients exhibit increased ameboidal type cell migration, axon development, blood vessel morphogenesis, circulatory system process, and collagen fibril organization. Furthermore, high-risk patients also undergo cellular processes such as cell division, proliferation, and formation of new cells, indicating a high rate of tumor cell division and proliferation in this patient group. These findings are consistent with the previous clinical conclusion that patients in the high-risk group generally have a poor prognosis. Using our 8-gene signature risk model, we investigated the KEGG functional pathways in high- and low-risk GC patients. Our analysis revealed that the five major pathways identified are generally associated with cancer development. Namely, cell adhesion molecules, complement and coagulation cascades, ECM receptor interaction, focal adhesion and hypertrophic cardiomyopathy were high in the high-risk patients (Francavilla et al., 2009; Jurcak et al., 2019; Yang et al., 2020; Zhang et al., 2020).

Moreover, high-risk patients showed a higher population of macrophages and neutrophils. Tumor-associated macrophages and neutrophils are generally associated with a poor prognosis in GC patients (Li et al., 2019; Gambardella et al., 2020). Macrophages are present at all stages of tumor progression at the primary site and are closely associated with tumor cell invasion (Nielsen and Schmid, 2017).

In addition, the high-risk group exhibited lower APC co-inhibitory effect scores than the low-risk group, indicating weakened antitumor immunity, which may contribute to poor prognosis. Tumor m6A research has recently gained attention, and the levels of FTO, METTL3, and YTHDC1 were significantly higher in the high-risk group compared to the low-risk group. FTO plays a critical role in the progression and metastasis of GC and is associated with low differentiation, lymph node metastasis, TNM stage, and poor prognosis, making it an important molecular marker for monitoring GC (Xu L et al., 2017). Yue et al. (2019) found that overexpression of METTL3 is associated with a poor prognosis in GC patients and promotes epithelial-mesenchymal transition and metastasis *in vivo*. Another study analyzed various biological information from different human cancer databases and discovered that YTHDF1 mutations are present in approximately 7% of GC patients. Elevated YTHDF1 levels are linked with increased cancer proliferation, invasiveness, and poorer overall survival in patients (Pi et al., 2021).

The KM survival curve also confirmed that differences in gene expression levels have varying effects on patient prognosis, with most patients exhibiting poor survival times when the gene expression is high. n addition, we predicted drugs that are closely related to the gene expression of our hub genes in order to explore their therapeutic effects on tumors. Positive correlation indicates that high expression of hub genes in GC is directly proportional to drug sensitivity. Negative correlation indicates that high gene expression in GC may affect drug efficacy. This study is the first to develop a prognostic model involving focal adhesion in GC patients, which was validated as an excellent predictor of patient overall survival (OS). Moreover, our model provides additional insights into immune infiltration, immune checkpoint markers, and pathway enrichment in different subgroups.

Although the model was validated in various aspects, there are still some limitations that need to be addressed. Firstly, the data used in this study were obtained from the TCGA and GEO databases, and thus, the generalizability of the model to other patient cohorts needs further validation. Additionally, further investigation is required to determine if the genes in the model act synergistically to influence GC patient prognosis. This study investigated the prognostic relevance of focal adhesion genes in GC patients using retrospective analysis. However, its predictive ability needs to be tested in prospective studies to validate its clinical application. Unlike traditional biological research methods, this method was based on a large dataset and possessed the advantages of enhanced efficiency, flexibility, and pertinence. With the continuous development of sequencing technology, this model has the potential for clinical application.

## Conclusion

In this study, we developed a prognostic model based on the focal adhesion genes COMP, FLNC, ITGB5, LAMC1, THBS1, THBS4, VEGFB, and VWF to differentiate clinical features and predict the prognosis of GC patients. Our results provide a valuable foundation and direction for future basic experimental and clinical research on GC.

## Data Availability

The datasets presented in this study can be found in online repositories. The names of the repository/repositories and accession number(s) can be found below: GSE13861, GSE29272 ,GSE62254 and GSE26942 (http://www.ncbi.nlm.nih.gov/geo/); TCGA-STAD (https://portal.gdc.cancer.gov/).

## Supplementary material

SUPPLEMENTARY FIGURE S1 Kaplan-Meier survival curves by the expression level of hub genes. **(A)** COMP; **(B)** FLNC; **(C)** ITGB5, **(D)** LAMC1, **(E)** THBS1, **(F)** THBS4, **(G)** VEGFB, **(H)** VWF. The patients were split into high and low expression groups according to the median value of the hub gene expression.

SUPPLEMENTARY FIGURE S2 Stratified analysis of the prognostic signature in the test cohort. The relationships between the FAS and age **(A)**, gender **(B)**, stage **(C)**.

SUPPLEMENTARY FIGURE S3 UMAP representation of the pooled cells depicted in different colours by **(A)** tissue and **(B)** sample. **(C)** The expression level of hub genes in the cell clusters of different tissues (gray indicates low, red indicates high).
