# Supplementary material for: Development and validation of focal adhesion-related genes signature in gastric cancer
Source: Front Genet. 2023 Mar 8;14:1122580. doi: 10.3389/fgene.2023.1122580 (PMC10030739; doi:10.3389/fgene.2023.1122580)
Supplement: Supplementary file 1 [file Table1.docx]

**Supplement Table 1**：The list of Focal adhesion related genes.

ACTB

ACTG1

ACTN1

ACTN2

ACTN3

ACTN4

AKT1

AKT2

AKT3

ARHGAP35

ARHGAP5

BAD

BCAR1

BCL2

BIRC2

BIRC3

BRAF

CAPN2

CAV1

CAV2

CAV3

CCND1

CCND2

CCND3

CDC42

CHAD

COL11A1

COL11A2

COL1A1

COL1A2

COL2A1

COL3A1

COL4A1

COL4A2

COL4A4

COL4A6

COL5A1

COL5A2

COL5A3

COL6A1

COL6A2

COL6A3

COL6A6

COMP

CRK

CRKL

CTNNB1

DIAPH1

DOCK1

EGF

EGFR

ELK1

ERBB2

FLNA

FLNB

FLNC

FLT1

FLT4

FN1

FYN

GRB2

GSK3B

HGF

HRAS

IBSP

IGF1

IGF1R

ILK

ITGA1

ITGA10

ITGA11

ITGA2

ITGA2B

ITGA3

ITGA4

ITGA5

ITGA6

ITGA7

ITGA8

ITGA9

ITGAV

ITGB1

ITGB3

ITGB4

ITGB5

ITGB6

ITGB7

ITGB8

JUN

KDR

LAMA1

LAMA2

LAMA3

LAMA4

LAMA5

LAMB1

LAMB2

LAMB3

LAMB4

LAMC1

LAMC2

LAMC3

MAP2K1

MAPK1

MAPK10

MAPK3

MAPK8

MAPK9

MET

MYL10

MYL12A

MYL12B

MYL2

MYL5

MYL7

MYL9

MYLK

MYLK2

MYLK3

MYLPF

PAK1

PAK2

PAK3

PAK4

PAK5

PAK6

PARVA

PARVB

PARVG

PDGFA

PDGFB

PDGFC

PDGFD

PDGFRA

PDGFRB

PDPK1

PGF

PIK3CA

PIK3CB

PIK3CD

PIK3CG

PIK3R1

PIK3R2

PIK3R3

PIK3R5

PIP5K1C

PPP1CA

PPP1CB

PPP1CC

PPP1R12A

PRKCA

PRKCB

PRKCG

PTEN

PTK2

PXN

RAC1

RAC2

RAC3

RAF1

RAP1A

RAP1B

RAPGEF1

RASGRF1

RELN

RHOA

ROCK1

ROCK2

SHC1

SHC2

SHC3

SHC4

SOS1

SOS2

SPP1

SRC

THBS1

THBS2

THBS3

THBS4

TLN1

TLN2

TNC

TNN

TNR

TNXB

VASP

VAV1

VAV2

VAV3

VCL

VEGFA

VEGFB

VEGFC

VEGFD

VTN

VWF

XIAP

ZYX
