# Supplementary figures and images for: Development and validation of focal adhesion-related genes signature in gastric cancer
Source: Front Genet. 2023 Mar 8;14:1122580. doi: 10.3389/fgene.2023.1122580 (PMC10030739; doi:10.3389/fgene.2023.1122580)

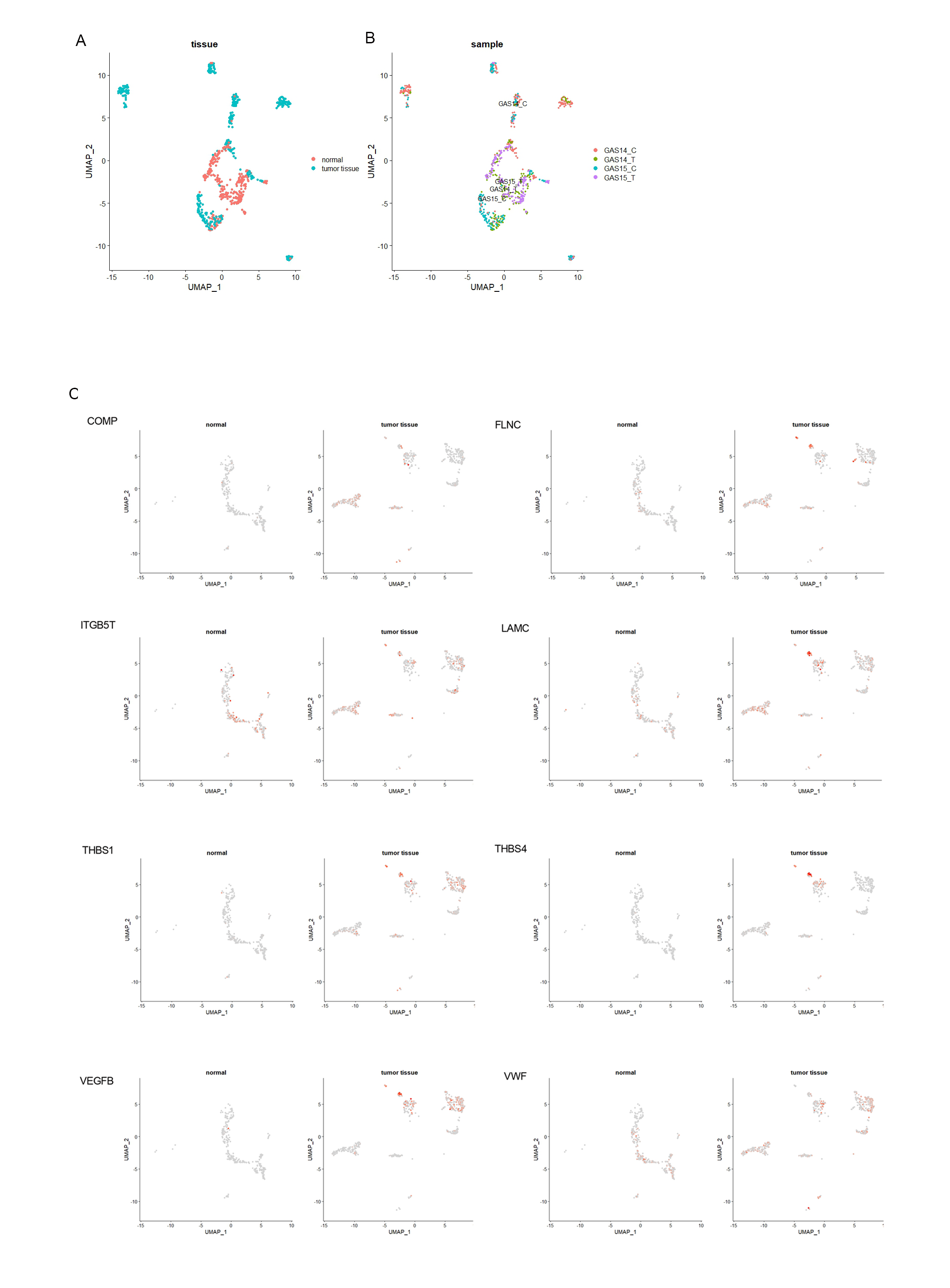

Supplement: Supplementary file 2 [file Image3.TIF]

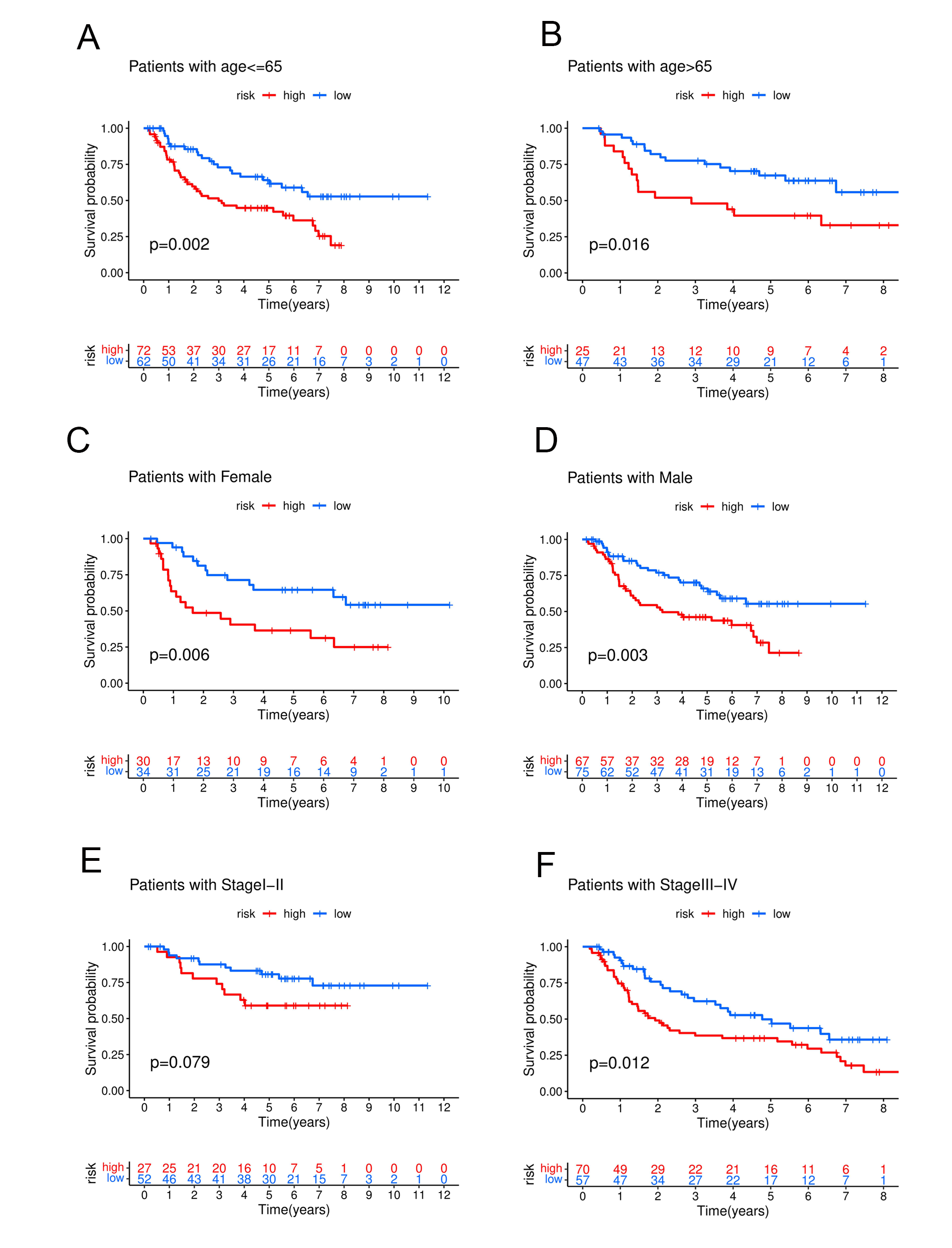

Supplement: Supplementary file 3 [file Image2.TIF]

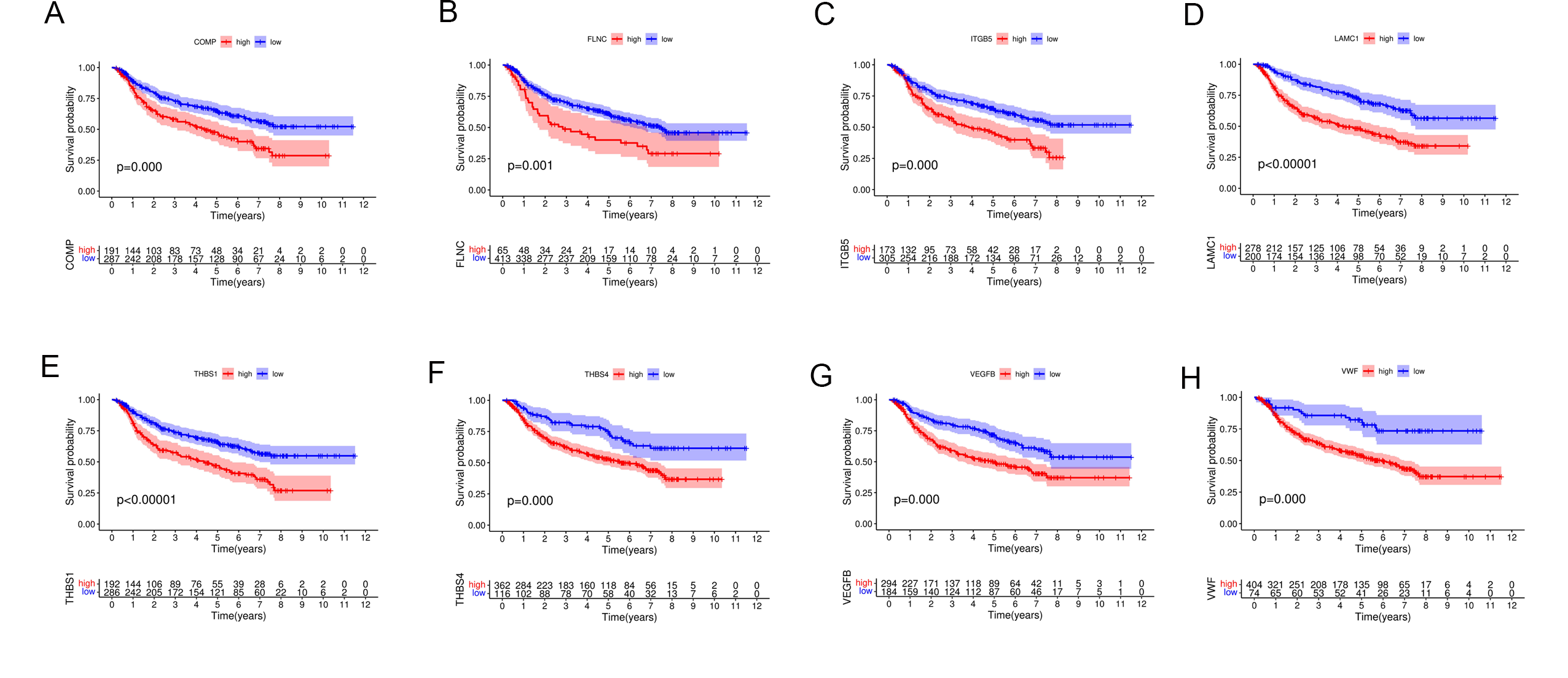

Supplement: Supplementary file 4 [file Image1.TIF]
