# Supplementary material for: Development and validation of focal adhesion-related genes signature in gastric cancer
Source: Front Genet. 2023 Mar 8;14:1122580. doi: 10.3389/fgene.2023.1122580 (PMC10030739; doi:10.3389/fgene.2023.1122580)
Supplement: Supplementary file 5 [file Table2.docx]

**Supplement Figure 2：** The correlation between the drugs and hub genes

| Gene | Drug | cor | pvalue |
| --- | --- | --- | --- |
| FLNC | Dasatinib | 0.623743 | 1.02E-07 |
| THBS1 | XAV-939 | 0.576782 | 1.41E-06 |
| LAMC1 | Palbociclib | -0.54311 | 7.34E-06 |
| FLNC | Staurosporine | 0.542286 | 7.62E-06 |
| THBS1 | Oxaliplatin | -0.53691 | 9.75E-06 |
| LAMC1 | AZD-8055 | 0.528697 | 1.41E-05 |
| LAMC1 | Ribavirin | -0.52435 | 1.71E-05 |
| LAMC1 | Barasertib | -0.5093 | 3.24E-05 |
| THBS1 | AMG-900 | -0.50726 | 3.53E-05 |
| THBS1 | Tamoxifen | -0.50425 | 3.99E-05 |
| THBS1 | By-Product of CUDC-305 | -0.50215 | 4.35E-05 |
| THBS1 | CUDC-305 | -0.5021 | 4.36E-05 |
| LAMC1 | Imexon | -0.49988 | 4.77E-05 |
| FLNC | JNJ-38877605 | 0.498454 | 5.05E-05 |
| LAMC1 | Oxaliplatin | -0.49637 | 5.50E-05 |
| LAMC1 | Dromostanolone Propionate | -0.49446 | 5.93E-05 |
| ITGB5 | Cyclophosphamide | -0.49386 | 6.07E-05 |
| ITGB5 | By-Product of CUDC-305 | -0.49023 | 7.01E-05 |
| ITGB5 | Oxaliplatin | -0.48261 | 9.42E-05 |
| LAMC1 | AMG-900 | -0.48245 | 9.48E-05 |
| LAMC1 | Dexrazoxane | -0.48201 | 9.64E-05 |
| LAMC1 | Irofulven | 0.475038 | 0.000125 |
| THBS1 | Everolimus | 0.472226 | 0.000139 |
| LAMC1 | tic10 | -0.47088 | 0.000146 |
| ITGB5 | ST-3595 | -0.47005 | 0.000151 |
| THBS1 | Staurosporine | 0.468881 | 0.000158 |
| ITGB5 | Chelerythrine | -0.46664 | 0.000171 |
| ITGB5 | XK-469 | -0.46408 | 0.000188 |
| LAMC1 | Crizotinib | -0.46088 | 0.000211 |
| LAMC1 | Volasertib | -0.4588 | 0.000227 |
| THBS1 | Volasertib | -0.45812 | 0.000233 |
| FLNC | DOLASTATIN 10 | -0.45542 | 0.000256 |
| ITGB5 | Imexon | -0.45318 | 0.000277 |
| THBS1 | Barasertib | -0.452 | 0.000288 |
| THBS1 | Ixabepilone | -0.44861 | 0.000324 |
| LAMC1 | XAV-939 | 0.447865 | 0.000333 |
| THBS1 | Crizotinib | -0.44765 | 0.000335 |
| FLNC | XAV-939 | 0.445781 | 0.000358 |
| THBS1 | Des-fluoro-TAK-960 | -0.44489 | 0.000369 |
| LAMC1 | TAK Plk inhibitor | -0.44404 | 0.00038 |
| LAMC1 | Cyclophosphamide | -0.4435 | 0.000387 |
| LAMC1 | Raloxifene | -0.44269 | 0.000397 |
| ITGB5 | AZD-8055 | 0.440936 | 0.000422 |
| ITGB5 | Dexamethasone Decadron | -0.43941 | 0.000444 |
| LAMC1 | CUDC-305 | -0.43799 | 0.000465 |
| ITGB5 | Nelarabine | -0.43608 | 0.000496 |
| FLNC | Simvastatin | 0.435377 | 0.000508 |
| LAMC1 | Tamoxifen | -0.4311 | 0.000585 |
| FLNC | Bleomycin | 0.429565 | 0.000615 |
| LAMC1 | By-Product of CUDC-305 | -0.42952 | 0.000615 |
| THBS1 | JNJ-38877605 | 0.429117 | 0.000624 |
| ITGB5 | Bendamustine | -0.4275 | 0.000657 |
| FLNC | BMS-690514 | 0.426197 | 0.000685 |
| LAMC1 | Deforolimius | 0.425477 | 0.000701 |
| THBS1 | TAK Plk inhibitor | -0.42479 | 0.000717 |
| LAMC1 | Ifosfamide | -0.42375 | 0.000741 |
| ITGB5 | Fenretinide | -0.4222 | 0.000779 |
| THBS1 | BP-1-102 | -0.42183 | 0.000788 |
| COMP | Thiotepa | 0.421215 | 0.000804 |
| COMP | Idarubicin | 0.419948 | 0.000837 |
| ITGB5 | AMONAFIDE | -0.41314 | 0.001035 |
| COMP | Triethylenemelamine | 0.412154 | 0.001067 |
| LAMC1 | Erlotinib | 0.411015 | 0.001106 |
| ITGB5 | DMAPT | -0.41076 | 0.001114 |
| LAMC1 | Des-fluoro-TAK-960 | -0.40944 | 0.00116 |
| ITGB5 | Hydroxyurea | -0.40789 | 0.001217 |
| THBS1 | AT-13387 | -0.40648 | 0.00127 |
| ITGB5 | Nitrogen mustard | -0.40645 | 0.001271 |
| ITGB5 | BN-2629 | -0.40633 | 0.001276 |
| ITGB5 | Kahalide F | 0.405057 | 0.001326 |
| FLNC | SB-590885 | -0.40505 | 0.001326 |
| THBS1 | Deforolimius | 0.404504 | 0.001348 |
| THBS1 | PX-316 | -0.40395 | 0.001371 |
| LAMC1 | AMONAFIDE | -0.40249 | 0.001432 |
| ITGB5 | Pyrazoloacridine | -0.40197 | 0.001454 |
| FLNC | Saracatinib | 0.400519 | 0.001519 |
| FLNC | EMD-534085 | -0.39947 | 0.001567 |
| THBS1 | Nilotinib | -0.39921 | 0.001579 |
| FLNC | Lenvatinib | 0.397722 | 0.00165 |
| FLNC | Midostaurin | 0.396208 | 0.001726 |
| ITGB5 | Melphalan | -0.3961 | 0.001731 |
| LAMC1 | DMAPT | -0.39573 | 0.00175 |
| LAMC1 | TAE-684 | -0.39477 | 0.0018 |
| THBS1 | TAK-960 analog | -0.39447 | 0.001816 |
| LAMC1 | GSK-2126458 | 0.390994 | 0.002009 |
| ITGB5 | Pipobroman | -0.39008 | 0.002063 |
| ITGB5 | FENRETINIDE | -0.39004 | 0.002065 |
| THBS1 | METHOTREXATE | -0.38909 | 0.002123 |
| THBS1 | Palbociclib | -0.38756 | 0.002218 |
| LAMC1 | Apitolisib | 0.386703 | 0.002273 |
| THBS1 | Erlotinib | 0.385594 | 0.002346 |
| ITGB5 | Ifosfamide | -0.38529 | 0.002366 |
| LAMC1 | Pyrazoloacridine | -0.38446 | 0.002422 |
| COMP | Valrubicin | 0.384246 | 0.002437 |
| ITGB5 | 8-Chloro-adenosine | -0.38391 | 0.00246 |
| ITGB5 | Arsenic trioxide | -0.38354 | 0.002486 |
| THBS1 | DOLASTATIN 10 | -0.38223 | 0.00258 |
| THBS1 | GSK-461364 | -0.38217 | 0.002585 |
| THBS1 | Homoharringtonine | -0.38122 | 0.002655 |
| COMP | Mitomycin | 0.380582 | 0.002702 |
| LAMC1 | Belinostat | -0.38006 | 0.002742 |
| THBS1 | TAK-901 | -0.37893 | 0.00283 |
| ITGB5 | DIGOXIN | -0.37854 | 0.002861 |
| LAMC1 | Chelerythrine | -0.37784 | 0.002917 |
| FLNC | SGX-523 | 0.376556 | 0.003023 |
| COMP | Bendamustine | 0.374597 | 0.00319 |
| LAMC1 | Imatinib | -0.37458 | 0.003192 |
| THBS1 | Vinblastine | -0.37343 | 0.003294 |
| ITGB5 | Chlorambucil | -0.37279 | 0.003352 |
| ITGB5 | Parthenolide | -0.3715 | 0.003473 |
| ITGB5 | Idarubicin | -0.37112 | 0.003509 |
| ITGB5 | Dexrazoxane | -0.37104 | 0.003516 |
| LAMC1 | Pevonedistat | -0.37085 | 0.003535 |
| ITGB5 | 3-Bromopyruvate (acid) | -0.37067 | 0.003552 |
| LAMC1 | Nelarabine | -0.37032 | 0.003586 |
| THBS1 | BI-2536 | -0.36962 | 0.003654 |
| LAMC1 | Staurosporine | 0.369183 | 0.003698 |
| FLNC | Zoledronate | 0.36836 | 0.003781 |
| LAMC1 | Hydroxyurea | -0.36788 | 0.00383 |
| THBS1 | Tanespimycin | -0.36771 | 0.003848 |
| LAMC1 | Asparaginase | -0.36743 | 0.003877 |
| LAMC1 | Elesclomol | -0.36738 | 0.003882 |
| LAMC1 | Fluphenazine | -0.3665 | 0.003975 |
| THBS1 | EMD-534085 | -0.36632 | 0.003994 |
| FLNC | LY-294002 | 0.36581 | 0.004049 |
| THBS1 | VINORELBINE | -0.36469 | 0.004172 |
| ITGB5 | Apitolisib | 0.364527 | 0.00419 |
| LAMC1 | Everolimus | 0.363156 | 0.004346 |
| VEGFB | Dovitinib | -0.36309 | 0.004354 |
| LAMC1 | Lomustine | -0.36291 | 0.004374 |
| THBS1 | Cyclophosphamide | -0.36277 | 0.00439 |
| THBS1 | BMS-690514 | 0.362502 | 0.004422 |
| FLNC | Sonidegib | 0.361902 | 0.004493 |
| ITGB5 | Methylprednisolone | -0.36164 | 0.004524 |
| LAMC1 | EMD-534085 | -0.36083 | 0.004622 |
| ITGB5 | Uracil mustard | -0.36037 | 0.004678 |
| LAMC1 | Nitrogen mustard | -0.3602 | 0.0047 |
| ITGB5 | Daunorubicin | -0.36011 | 0.004711 |
| LAMC1 | JNJ-38877605 | 0.360021 | 0.004721 |
| ITGB5 | Fludarabine | -0.35979 | 0.00475 |
| ITGB5 | XAV-939 | 0.359649 | 0.004768 |
| ITGB5 | Lomustine | -0.3592 | 0.004824 |
| THBS1 | Dromostanolone Propionate | -0.35857 | 0.004904 |
| THBS1 | Eribulin mesilate | -0.35815 | 0.004959 |
| LAMC1 | Carmustine | -0.35807 | 0.004968 |
| LAMC1 | Masitinib | -0.35784 | 0.004999 |
| LAMC1 | 6-THIOGUANINE | -0.35758 | 0.005032 |
| THBS1 | Sapitinib | 0.356981 | 0.005112 |
| THBS1 | Actinomycin D | -0.3566 | 0.005163 |
| FLNC | CCT-128930 | 0.355315 | 0.005338 |
| FLNC | Tamoxifen | -0.35518 | 0.005356 |
| ITGB5 | METHOTREXATE | -0.35499 | 0.005383 |
| VEGFB | ENMD-2076 | -0.35448 | 0.005454 |
| LAMC1 | DOLASTATIN 10 | -0.35413 | 0.005503 |
| FLNC | By-Product of CUDC-305 | -0.35255 | 0.005733 |
| LAMC1 | Sapitinib | 0.351456 | 0.005895 |
| ITGB5 | GSK-2126458 | 0.351106 | 0.005948 |
| VEGFB | Astex FGF inhibitor | -0.35099 | 0.005967 |
| ITGB5 | Carmustine | -0.35063 | 0.006021 |
| THBS1 | Rapamycin | 0.350221 | 0.006084 |
| COMP | Pevonedistat | 0.349737 | 0.00616 |
| LAMC1 | Ixabepilone | -0.3491 | 0.006261 |
| FLNC | 5-Fluoro deoxy uridine 10mer | 0.348609 | 0.006339 |
| LAMC1 | OSI-027 | 0.348336 | 0.006383 |
| LAMC1 | PYRAZOLOACRIDINE | -0.34773 | 0.006481 |
| VEGFB | E-7820 | -0.34756 | 0.00651 |
| LAMC1 | BP-1-102 | -0.3471 | 0.006586 |
| THBS1 | RAPAMYCIN | 0.347088 | 0.006588 |
| LAMC1 | TAK-960 analog | -0.34682 | 0.006632 |
| LAMC1 | OSU-03012 | -0.3466 | 0.00667 |
| THBS1 | Dasatinib | 0.344356 | 0.007056 |
| LAMC1 | Teglarinad | -0.3438 | 0.007155 |
| FLNC | Cediranib | 0.343139 | 0.007273 |
| COMP | Pipobroman | 0.342807 | 0.007334 |
| VEGFB | XR-5944 | -0.34248 | 0.007395 |
| THBS4 | Thiotepa | 0.341394 | 0.007596 |
| COMP | XK-469 | 0.341022 | 0.007666 |
| LAMC1 | BI-2536 | -0.34077 | 0.007715 |
| LAMC1 | PF-04691502 | 0.340709 | 0.007726 |
| LAMC1 | Calusterone | -0.34068 | 0.007732 |
| LAMC1 | BMS-690514 | 0.339727 | 0.007916 |
| THBS4 | RAPAMYCIN | -0.33956 | 0.007948 |
| ITGB5 | Ribavirin | -0.33901 | 0.008057 |
| THBS1 | Luminespib | -0.33875 | 0.008109 |
| COMP | Hydroxyurea | 0.33813 | 0.008233 |
| LAMC1 | ABT-737 | -0.33735 | 0.008393 |
| LAMC1 | AZD-5363 | 0.336911 | 0.008482 |
| LAMC1 | Pictilisib | 0.336906 | 0.008483 |
| COMP | OSI-027 | -0.33675 | 0.008515 |
| ITGB5 | Etoposide | -0.3365 | 0.008569 |
| COMP | Teniposide | 0.336342 | 0.008601 |
| COMP | Irinotecan | 0.335982 | 0.008677 |
| ITGB5 | LOR-253 | -0.33472 | 0.008947 |
| LAMC1 | Mocetinostat | -0.3344 | 0.009017 |
| THBS1 | Belinostat | -0.33426 | 0.009047 |
| FLNC | TYROTHRICIN | -0.33378 | 0.009154 |
| ITGB5 | Lapachone | -0.33366 | 0.009179 |
| ITGB5 | LMP776 | -0.33322 | 0.009278 |
| LAMC1 | XK-469 | -0.33298 | 0.009333 |
| ITGB5 | Palbociclib | -0.33288 | 0.009354 |
| ITGB5 | RH1 | -0.33257 | 0.009425 |
| LAMC1 | SR16157 | -0.33151 | 0.009668 |
| FLNC | Vemurafenib | -0.3314 | 0.009694 |
| ITGB5 | Cytarabine | -0.33131 | 0.009714 |
| LAMC1 | Isotretinoin | -0.33031 | 0.009952 |
